# A network pharmacology approach to reveal the pharmacological targets and biological mechanism of compound kushen injection for treating pancreatic cancer based on WGCNA and in vitro experiment validation

**DOI:** 10.1186/s13020-021-00534-y

**Published:** 2021-11-22

**Authors:** Chao Wu, Zhi-Hong Huang, Zi-Qi Meng, Xiao-Tian Fan, Shan Lu, Ying-Ying Tan, Lei-Ming You, Jia-Qi Huang, Antony Stalin, Pei-Zhi Ye, Zhi-Shan Wu, Jing-Yuan Zhang, Xin-Kui Liu, Wei Zhou, Xiao-Meng Zhang, Jia-Rui Wu

**Affiliations:** 1 School of Chinese Materia Medica, Beijing University of Chinese Medicine, Beijing, 102488 China; 2 School of Life Science, Beijing University of Chinese Medicine, Beijing, 102488 China; 3 State Key Laboratory of Subtropical Silviculture, Department of Traditional Chinese Medicine, Zhejiang A&F University, Hangzhou, 311300 China; 4 National Cancer Center/National Clinical Research Center for Cancer/Chinese Medicine Department of the Caner Hospital, Chinese Academy of Medical Sciences and Peking Union Medical College, Beijing, China; 5 China-Japan Friendship Hospital, Beijing, 100029 China

**Keywords:** Compound kushen injection, Pancreatic cancer, WGCNA, Network pharmacology

## Abstract

**Background:**

Compound kushen injection (CKI), a Chinese patent drug, is widely used in the treatment of various cancers, especially neoplasms of the digestive system. However, the underlying mechanism of CKI in pancreatic cancer (PC) treatment has not been totally elucidated.

**Methods:**

Here, to overcome the limitation of conventional network pharmacology methods with a weak combination with clinical information, this study proposes a network pharmacology approach of integrated bioinformatics that applies a weighted gene co-expression network analysis (WGCNA) to conventional network pharmacology, and then integrates molecular docking technology and biological experiments to verify the results of this network pharmacology analysis.

**Results:**

The WGCNA analysis revealed 2 gene modules closely associated with classification, staging and survival status of PC. Further CytoHubba analysis revealed 10 hub genes (*NCAPG, BUB1, CDK1, TPX2, DLGAP5, INAVA, MST1R, TMPRSS4, TMEM92* and *SFN*) associated with the development of PC, and survival analysis found 5 genes (*TSPOAP1, ADGRG6, GPR87, FAM111B* and *MMP28*) associated with the prognosis and survival of PC. By integrating these results into the conventional network pharmacology study of CKI treating PC, we found that the mechanism of CKI for PC treatment was related to cell cycle, JAK-STAT, ErbB, PI3K-Akt and mTOR signalling pathways. Finally, we found that *CDK1, JAK1, EGFR, MAPK1* and *MAPK3* served as core genes regulated by CKI in PC treatment, and were further verified by molecular docking, cell proliferation assay, RT-qPCR and western blot analysis.

**Conclusions:**

Overall, this study suggests that the optimized network pharmacology approach is suitable to explore the molecular mechanism of CKI in the treatment of PC, which provides a reference for further investigating biomarkers for diagnosis and prognosis of PC and even the clinical rational application of CKI.

**Supplementary Information:**

The online version contains supplementary material available at 10.1186/s13020-021-00534-y.

## Introduction

Pancreatic cancer (PC) is a common malignant tumour of the digestive tract characterized by concealed onset, high malignancy, and rapid development, and the majority of PC cases have locally advanced or metastatic at the time of diagnosis [1–3]. According to Global Cancer Statistics, approximately 466,000 deaths (4.7% of total cases) worldwide in 2020 are attributable to PC, which ranks seventh in cancer-related mortality [4]. In countries with a high human development index, such as Europe and North America, the incidence of PC is three to four times higher, and the number of deaths from PC in the United States is expected to increase significantly by 2030, which PC will become the second leading cause of cancer death [4, 5]. PC is one of the worst prognostic tumours among all malignant tumours. Tumour metastasis occurs in about 60% of PC patients when diagnosed. The median survival time of patients is only 6–15 months, and the five-year survival rate is lower than 6%. Human health and life are under severe constant threat of PC [6].

Compound kushen injection (CKI), is made of Kushen (Radix Sophorae flavescentis) and Baituling (Rhizoma Smilacisglabrae) by modern technology [7], has been approved by China Food and Drug Administration (CFDA) in 1995 as an adjuvant drug for cancer treatment, which can relieve pain, activate innate immune response and reduce side effects in cancer treatment [8, 9]. Moreover, CKI has been found to inhibit the tumour cell growth, proliferation, metastasis and invasion, induce tumour cell apoptosis, and have the functions of anti-multidrug resistance and protection of human immune function [10]. The combination of CKI and chemo(radio)therapy improves the therapeutic effect and quality of life in PC patients. In addition, CKI has been reported a direct inhibitory effect on human PC cells SW1990 in vitro [11, 12]. However, the anti-PC effect of CKI has been confirmed while the underlying molecular mechanism is still a mystery.

Network pharmacology has become an effective tool to elucidate the complex overall mechanism of traditional Chinese medicine (TCM) and provides a new perspective for analyzing drug effects [13]. In accordance with the TCM characteristics of multi-component, multi-target, multi-pathway synergy, network pharmacology transforms the "one target, one drug" model into a new "multi-target, multi-component" model and clarifies the complex interactions among genes, proteins, and metabolites related to diseases and drugs from a network perspective [14, 15]. But, the limitations such as the clinical information deficiency restrict the value and application of network pharmacology. Zhang and Horvath first developed weighted gene co‐expression network analysis (WGCNA) in 2005, which has become a standard algorithm used for gene co-expression network construction [16, 17]. Currently, WGCNA is used in several studies of complex human diseases [18–20], especially in cancers, such as lung cancer [21], breast cancer [22], cholangiocarcinoma [23], etc. Benefiting from it is special function, which can discover modules of highly correlated genes and correlate with modules and external sample characteristics (i.e., associated key genes with clinical features), WGCNA is generalized to various research areas [24]. It conferred more clinical significance to its findings. Coincidentally, this also exactly compensates for the shortage of clinical information loss in network pharmacology.

**Fig. 1:**
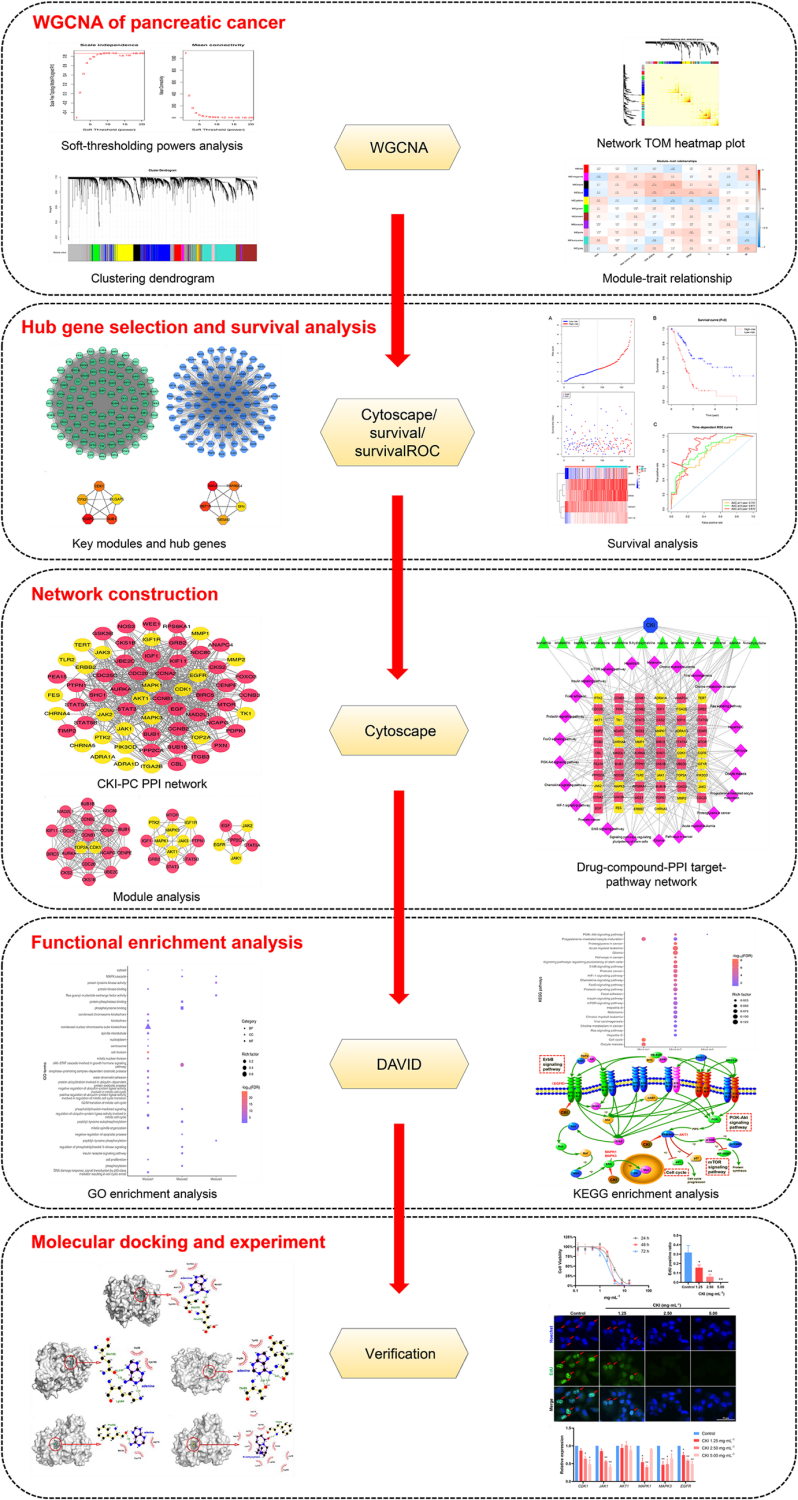
The flowchart of network pharmacology approach used in this study for exploring the molecular mechanism of CKI treating PC

In the present study, we aim to apply WGCNA to optimize network pharmacology methods. Firstly, WGCNA was used to construct a gene co-expression network, find clusters (modules) of highly related genes, modules and correlate with external sample traits. And then, we screened the relevant modules to obtain biomarkers that were closely related to PC development. Survival analyses were employed to identify hub genes affecting the pathogenesis and prognosis of PC patients. In addition, we constructed a “compound-putative target network”, “CKI-PC The protein–protein interaction (PPI) network”, “drug-compound-PPI target-pathway network” through the network pharmacology method to explore the mechanism of CKI in the treatment of PC. This study aimed to reveal the complex mechanism of multi-component, multi-target, and multi-pathway of CKI in the treatment of PC at a system level, and provide a better basis for the diagnosis, treatment and prognosis of PC. Figure 1 shows a flowchart of the network pharmacology approach used in this study.

## Materials and methods

### Data collection and preprocessing

RNA sequencing data of pancreatic adenocarcinoma, containing a total of 182 samples, were obtained from The Cancer Genome Atlas (TCGA) database [25]. After removing non-cancerous samples and metastatic tumour samples, 177 primary tumour samples were obtained for further analysis. The “goodSamplesGenes” function in the WGCNA package was utilized to delete the samples that had too many missing values [24]. Also, the “hclust” function was used to perform hierarchical cluster analysis, and the outliers were all deleted. Finally, we selected the top 5000 genes that are most important for differential expression for the following WGCNA analysis. Meanwhile, the clinical metadata of 177 samples was also downloaded from the TCGA database and filtered for useful information (such as race, age, vital status, grade, stage and so on).

### Construction of co-expression network

The WGCNA package was used to construct the gene co-expression networks. To ensure that the connections between genes match the scale-free network distribution, the WGCNA algorithm selects the results that best fit the scale-free network distribution by choosing the weighting parameters. The soft threshold power β selected by the “pickSoftThreshold” function was used to achieve scale-free topology. The soft threshold power β refers to the correlation coefficient between the logarithmic value of the node with a connection degree k (log(k)) and the logarithmic value of the probability of a node with a connection degree k (log(p(k))).

### Identify gene modules and correlate clinical information

In this study, the topological overlap matrix was reconstructed by computing topological overlap measure (TOM), which is a robust measure of network interconnectedness. According to the dissimilarity matrix, which represents the connection relationship of genes, cluster analysis of genes by dissimilarity was performed to construct a hierarchical cluster tree [26]. The dynamic tree-cut algorithm method was adopted to identify the module of gene co-expression with values maxBlockSize = 6000, minModuleSize = 30 and mergeCutHeight = 0.2. Module eigengene (ME) refers to the first principal component of each gene module, and the expression of ME is considered to represent all genes in a module. The most important module can be found by calculating the correlation coefficient between ME and the clinical traits of interest. Gene significance (GS) indicates the degree of correlation between individual genes and clinical information. For each module, module membership (MM) is defined as the correlation between a single gene and ME, used to measure the importance of this gene in the module. According to ME, GS and MM, we can associate modules with clinical traits, not only to calculate the correlation between ME and clinical traits, but also to analyze clinically important modules.

### Identify hub genes and external validation

CytoHubba [27], a plug-in of Cytoscape software [28], is available for exploring important nodes of biological networks. In this study, Cytoscape was employed to visualize the module network, and CytoHubba was used to analyze the network. The maximal clique centrality (MCC) algorithm was then selected to determine the top 5 genes with the highest scores among the important modules as hub genes.

Then, we selected the data in the GEO database as the external validation dataset. We searched the GEO database using the term “pancreatic cancer” and the criteria for inclusion of PC chip datasets were to meet the following: ① Patients newly diagnosed with pancreatic cancer and not receiving any treatment; ② Datasets with total samples greater than 50. We performed differential expression analysis after normalizing and homogenizing raw data using the limma package in R, followed by Benjamini & Hochberg (false discovery rate) correction of *p*-values.

### Survival analysis

In this study, a univariate Cox proportional hazards regression analysis was performed for the genes in the selected modules using the survival package to identify candidate genes strongly correlated with survival. Subsequently, candidate genes that screened according to *P*-value were entered into the multivariate Cox proportional hazards regression analysis. Based on the selected gene expression profile, a survival-related linear risk assessment model was constructed. The formula for calculating the prognostic risk score is:

$${\text{risk score}} = \mathop \sum \limits_{{{\text{i}} = 1}}^{{\text{n}}} \left( {{\text{exp}}_{{\text{i}}} \times {\text{coef}}_{{\text{i}}} } \right)$$

Using the median prognostic risk score as a cut-off value, samples were divided into high- and low-risk groups. Furthermore, the correlation between gene expression and survival time was confirmed by calculating the hazard ratio (HR) and 95% confidence interval (CI). The Kaplan–Meier and Log-rank test methods were used to evaluate the differences in overall survival rates between the high- and low-risk groups. The survivalROC package analyzed a time-dependent receiver operating characteristic curve (ROC curve) to evaluate the prediction accuracy of the prognostic signature for time-dependent cancer death. Moreover, the predictive ability of the gene signature for clinical outcomes was measured by calculating the area under the curve (AUC).

### Identification of CKI compounds and targets

CKI (batch number: 20200329) was supplied by Zhendong Pharmaceutical Co. Ltd (Shanxi, China). Shimadzu Nexera LC-40-QE-Orbitrap-MS Separation was performed on an analytical column of Hypersil BDS (150 mm × 4. 6 mm, 5 μm). The oven was set at 25 ℃; The injection volume was 5 μL; The flow rate was set at 0.5 mL min^−1^; The mobile phase was consisted of 0.1% ammonia in water (A) and carbinol (B). The eluting program was: 5–20% B for 0–1 min, 20–80% B for 1–30 min, 80–60% B for 30–60 min. The ion source was electrospray ionization (ESI); MS was operated in positive/negative mode; The scan mode was Full scan/ddMS2; The scan range was 100–1200 Da; The capillary temperature was 350 ℃; The spray voltage in negative mode was 3800 V; The spray voltage in positive mode was 3200 V; The sheath gas was 35 arb; the aux gas was 15 arb; Three collision energies of low, medium and high were used for MS2. The positive ion mode was 30 eV, 40 eV, 50 eV, and the negative ion mode was 30 eV, 50 eV, 70 eV. The resolution of the Full scan was 70,000 FWHM, and the resolution of MS2 was 17,500 FWHM. The reference marker compounds present in the sample were identified based on retention time, MS fragmentation and UV spectra.

Then, the structural information of the CKI compounds was retrieved from the PubChem database [29] (https://pubchem.ncbi.nlm.nih.gov/). The simplified molecular input line entry specification (SMILES) of these compounds were imported into four databases, Search Tool for Interactions of Chemicals (STITCH) [30], SuperPred [31], SwissTargetPrediction [32], and Traditional Chinese Medicine System Pharmacology Database and Analysis Platform (TCMSP) [33], to collect the known and predicted human targets of these compounds.

### Collection of PC targets

The human genes associated with PC were obtained from three resources: (1) 10 hub genes related to the occurrence and development of PC and 5 potential biomarkers closely related to prognosis obtained from WGCNA; (2) Therapeutic Target Database (TTD) database [34]. The TTD database was searched using the key word "pancreatic cancer"; (3) TCGA database. RNA sequencing data of pancreatic adenocarcinoma were obtained from the TCGA database.

Data were normalized and analyzed by the edgeR package [35]. Differential expression genes (DEGs) were then screened out using | log_2_FC |> 1 and adjust *P* < 0.05. The PPI data were extracted from the Search Tool for the Retrieval of Interacting Genes (STRING, https://stringdb.org/) database [36]. The PPI data with the species limited to “*Homo sapiens*”, and the confidence score greater than 0.7 (high confidence) were selected for further investigation.

### Network construction

In this study, we constructed three networks: (1) compound-putative target network was built by connecting CKI compounds and their related targets; (2) CKI-PC PPI network was constructed by connecting the intersections of the compound targets and PC-related targets and other human proteins interacting with them; (3) drug-compound-PPI target-pathway network was constructed by connecting CKI, compounds, PPI targets, and related pathways. The above networks were visualized using Cytoscape and the topological features of the interaction networks were evaluated by calculating three indices (degree, betweenness, closeness) using the NetworkAnalyzer plug-in.

### Module analysis and enrichment analysis

To identify the core clustering module in the CKI-PC PPI network, the plug-in Molecular Complex Detection (MCODE) in Cytoscape was used for module analysis. DAVID (https://david.ncifcrf.gov/, version 6.8) [37] was employed to perform Gene Ontology (GO) function enrichment and Kyoto Encyclopedia of Genes and Genomes (KEGG) pathway enrichment analysis for the selected modules. The significance threshold was set at *P* < 0.05 and the false discovery rate (FDR) was set at < 0.05.

### Molecular docking

The three-dimensional crystal structures of the core target were extracted from the Research Collaboratory for Structural Bioinformatics (RCSB) Protein Database [38] (PDB, https://www.RCSB.org/). Then, the protein structures were processed by AutoDock Tools (ADT) [39], including removal of ligands and water molecules, calculation of Gasteiger charge, addition of polar hydrogen, and combination of non-polar hydrogen. ADT was also utilized to prepare corresponding compounds. Subsequently, molecular docking was carried out via AutoDock Vina [40]. Finally, the receptor-ligand complex was imported into Ligplus software to analyze the hydrogen bonding and hydrophobic interaction between the receptor and ligand.

### Cell lines and cell proliferation assays

Human PC cell lines Panc-1 was purchased from Procell Life Science &Technology Co., Ltd. (Wuhan, China), and cultured in Dulbecco’s modified Eagle’s medium (DMEM, Corning, USA) containing 10% fetal bovine serum (Corning, USA) and 1% penicillin/streptomycin (Gibco, USA) in a saturated humidity environment at 37 °C and 5% CO2. For CKI incubation [41], CKI (total alkaloid concentration of 25 mg mL^−1^) was diluted with DMEM (CKI concentrations: 0.125, 0.25, 0.5, 1.0, 2.0, 4.0, 8.0, 16.0 mg mL^−1^, using the doubling dilution method, based on the total alkaloid concentration in CKI).

The proliferation of Panc-1 cells was detected by Cell Counting Kit-8 (CCK-8, Dojindo, Japan) assay and 5‐ethynyl‐20-deoxyuridine (EdU) proliferation assay. In brief, after routine digestion, cells were blown into a single-cell suspension (2.0 × 10^5^ cells mL^−1^) and seeded into 96-well plates (100 μL·well^−1^), and cultured for 24 h, routinely. The cells were then cultured with a drug-containing medium for 24, 48 and 72 h respectively. After drug treatment, the CCK-8 solution was added into 96-well plates (10 μL·well^−1^) and incubated for 4 h at 37 °C. Optical density (OD) was detected at 450 nm using a microplate reader (Molecular Devices, USA). EdU proliferation assay was performed by BeyoClick™ EdU Cell Proliferation Kit with Alexa Fluor 488 (Beyotime, China) and strictly followed the manufacturer's protocol.

### Reverse transcription quantitative polymerase chain reaction (RT-qPCR) analysis

RNA Easy Fast Cell Kit (Tiangen, China) was applied for total RNA isolation according to the manufacturer’s instruction. The quality of total RNA was accredited by SpectraMax Quick Drop readers (Molecular Devices, USA). Of the total RNA, 1 μg was used for cDNA synthesis following the ReverTra Ace qPCR RT Kit (Toyobo, Japan) instruction. RT-qPCR was performed to measure the relative expression of mRNA, using SYBR Green Realtime PCR Master Mix (Toyobo, Japan). GAPDH was used as a control and the 2^−ΔΔCt^ method was conducted for the data analysis. The primer sequence of target genes was synthesized by Sangon Biotech Co., Ltd (Shanghai, China, Table 1).

**Table 1:** qPCR primer sequence

Gene		5' to 3'
AKT1	Forward	TCTATGGCGCTGAGATTGTG
	Reverse	CTTAATGTGCCCGTCCTTGT
CDK1	Forward	TAGGCGGGATCTACCATACCC
	Reverse	TCATGGCTACCACTTGACCTG
EGFR	Forward	TGTGCCCACTACATTGACGG
	Reverse	TAGGCCCATTCGTTGGACAG
JAK1	Forward	AGGGGATGGACTATTTGGGTTCTC
	Reverse	CCTTATCGGTTTCAATTGCTTTGG
MAPK1	Forward	GGAACTATTTGCTTTCTCTTCC
	Reverse	CTACTTCAATCCTCTTGTGTGG
MAPK3	Forward	ATCAACACCACCTGCGACCTTA
	Reverse	TACCAGCGCGTAGCCACATACT

### Enzyme-linked immunosorbent assay (ELISA)

Human AKT1 ELISA Kit, Human CDK1 ELISA Kit, Human JAK1 ELISA Kit, Human EGFR ELISA Kit, Human MAPK1 ELISA Kit and Human MAPK3 ELISA Kit (Sinobestbio, China) were used to detect the protein expression of 6 core targets following the manufacturer's instructions.

### Western blot assay

Panc-1 cells were collected in RIPA lysis buffer and centrifuged at 13,000 rpm and 4 ℃ for 10 min. The supernatants were preserved and used for western blot assay. Total protein concentration was gauged by BCA Protein Assay Kit (Solarbio, China). 20 µg of total protein was mixed with 5 × sample buffer, boiled at 99 ℃ for 5 min and loaded onto 10% SDS-PAGE gels. Then the protein bands were transferred onto NC membranes and blocked with 5% non-fat milk or 5% bovine serum albumin (BSA) for 2 h at room temperature. The NC membranes with proteins were incubated with diluted primary antibodies (Affinity or Proteintech, China) at 4 ℃ overnight, including anti-AKT1 (1:500), anti-phospho-AKT1 (p-AKT1, 1:500), anti-CDK1 (1:500), anti-phospho-CDK1 (p-CDK1, 1:1000), anti-JAK1 (1:500), anti-phospho-JAK1 (p-JAK1, 1:500), anti-EGFR (1:500), anti-phospho-EGFR (p-EGFR, 1:500), anti-MAPK3/MAPK1 (1:500), anti-phospho-MAPK3/MAPK1 (p-MAPK3/1, 1:500) and anti-β-Tubulin (1:2000) antibodies. Then, membranes were incubated with relative sources of secondary antibodies (1:5000) at room temperature for 1.5 h. At last, the specific protein bands were recognized with immobilon western chemiluminescent HRP substrate (MilliporeSigma, USA). Image J software was used for image analysis and the signals of specific proteins were normalized to β-Tubulin.

### Statistical analysis

Data were presented as mean ± SD and statistical analysis was performed with the two-tailed unpaired Student’s t-test using GraphPad Prism 9.0 software. In all statistical analyses, statistical significance was indicated by a single asterisk (*: *P* < 0.05), two asterisks (**: *P* < 0.01).

## Results

### WGCNA module construction

**Fig. 2:**
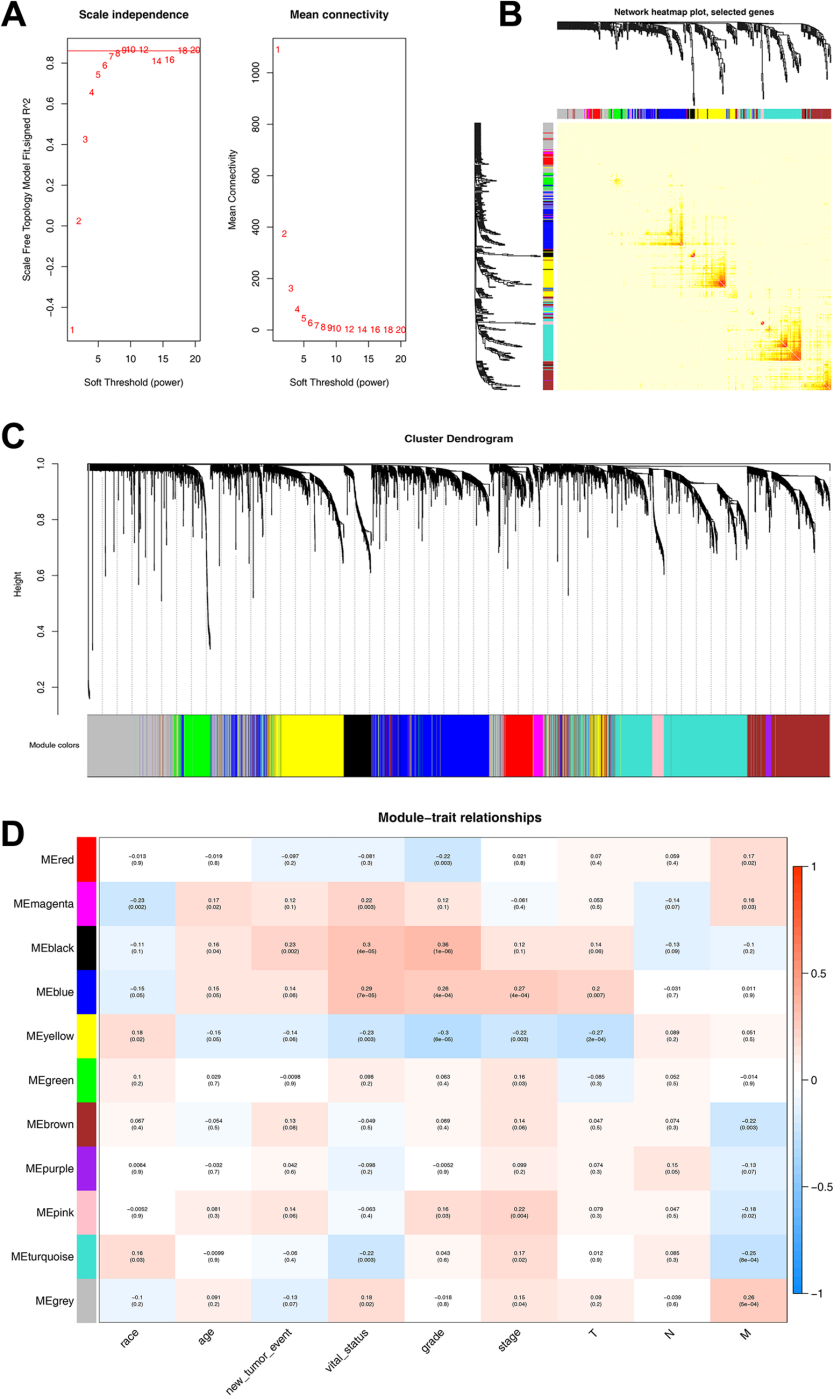
Construction of co-expression module. **A** Soft-thresholding powers analysis. R2 = 0.85. **B** Cluster diagram of gene modules. Different colors represent different gene modules, and gray modules are composed of genes that do not belong to any module. **C** Network TOM heatmap plot. TOM plot was made up by randomly selected 400 genes. Each row and column represented a module and the genes of the module. This diagram showed the degree of correlation within the module. **D** Module–trait relationship. Each row corresponds to a ME, and each column corresponds to a clinical trait. Each cell contains a corresponding correlation and *P*-value of modules with various clinical traits

In this study, a total of 177 samples and 5000 genes were screened for WGCNA analysis. All samples were retained as no outliers were found in the cluster analysis of the samples. In this study, a power value of β = 9 (R^2^ = 0.85) was selected according to the scale-free criterion to construct the gene co-expression network (Fig. 2A). Then, a hierarchical clustering tree was built, and gene modules were identified using the dynamic tree cut method. The minimum number of genes in each module was set to 30. Similar expression modules were merged and 11 modules were obtained (Fig. 2B). In addition, the TOM was visualized with a heatmap that could depict adjacencies or topological overlaps (Fig. 2C).

### Correlation analysis between modules and clinical traits

The results and discussion may be presented separately or in a combined section and can optionally be divided into headed subsections. In this study, we calculated the correlation between gene modules and clinical traits such as race, age, new tumour event, vital status, grade, and stage of patients with PC. The Pearson correlation coefficient of ME and the corresponding variables was employed to represent the correlation between modules and the corresponding clinical information. As shown in Fig. 2D, the correlation between the black module and grade was greater than other modules, and the correlation between the blue module and stage was greater than other modules. Therefore, these two modules were selected for further investigation.

### Hub genes screening

**Fig. 3:**
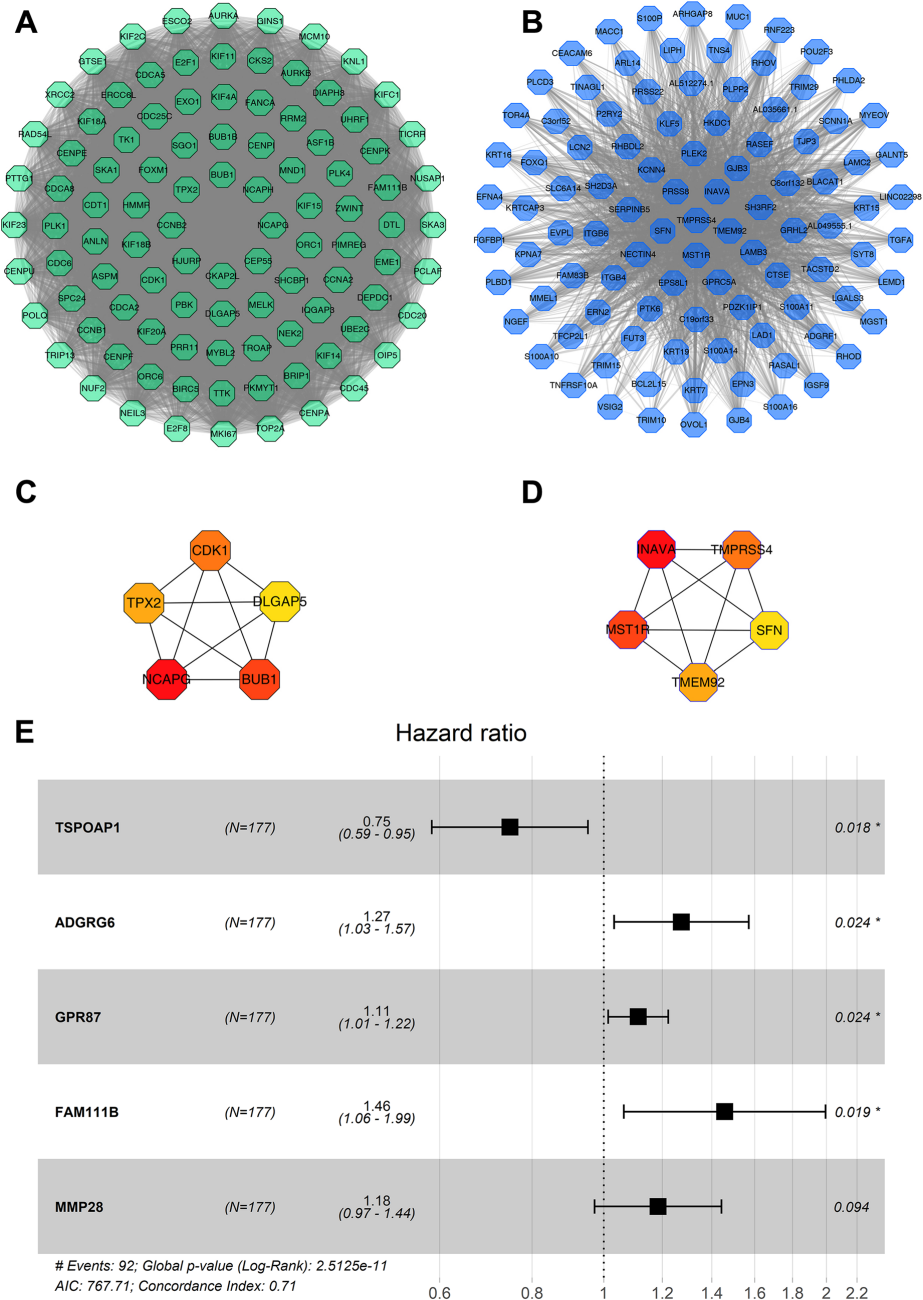
Modular gene network and Cox regression analysis. **A** The top 100 genes network in the black module. **B** The top 100 genes network in the blue module. **C** 5 hub genes selected by the black module. **D** 5 hub genes selected by the blue module. The redder the color, the higher the MCC score. **E** Forest plot of multivariate Cox regression analysis

When screening the network of black and blue gene modules with the weight of Cutoff = 0.1 as the threshold, the black module consisted of 6173 gene linkages and 132 genes, and the blue module consisted of 4145 gene linkages and genes. Cytoscape was used to visualize the top 100 genes in the black and blue modules respectively (Fig. 3A, B). In addition, CytoHubba was used to analyze the network to determine the top 5 genes of the MCC score as hub genes (Fig. 3C, D). The hub genes of the black module are: non-SMC condensin I complex subunit G (NCAPG), mitotic checkpoint serine/threonine-protein kinase BUB1 (BUB1), cyclin-dependent kinase 1 (CDK1), targeting protein for Xklp2 (TPX2) and disks large-associated protein 5 (DLGAP5). The hub genes of the blue module are: innate immunity activator protein (INAVA), macrophage-stimulating protein receptor (MST1R), transmembrane protease serine 4 (TMPRSS4), transmembrane protein 92 (TMEM92) and 14-3-3 protein sigma (SFN).

### Survival analysis

The univariate Cox proportional hazards regression analysis was used to study the correlation between mRNAs selected in the black and blue modules and survival time. Twenty-four genes related to survival time were selected based on *P* < 5E−06. The results of the univariate Cox proportional hazards regression are shown in Table 2. In addition, the results of multivariate Cox proportional hazards regression analysis showed that one gene (*TSPOAP1*) with HR < 1 was identified as a protective prognostic gene (positively correlated with the survival time of the patient); four genes (*ADGRG6, GPR87, FAM111B, MMP28*) with HR > 1 were identified as risky prognostic genes (inversely associated with the survival time of patients, Fig. 3E). The regression coefficients of the multivariate Cox proportional hazards regression analysis of the 5 genes were extracted to construct a prognostic risk scoring model:

$$\begin{aligned} {\text{Risk score }} = & \, \left( { - 0.{292 } \times TSPOAP1\;{\text{expression}}} \right) \, + \, \left( {0.{242 } \times ADGRG6\;{\text{expression}}} \right) \, \\ & + \, \left( {0.{1}0{7 } \times GPR87\;{\text{expression}}} \right) \, + \, \left( {0.{376 } \times FAM111B\;{\text{expression}}} \right) \, + \, \left( {0.{169 } \times MMP28\;{\text{expression}}} \right) \\ \end{aligned}$$

**Table 2:** Preliminary screening of single-factor Cox proportional hazard model

Gene	Coef	Exp (coef)	z	*P* value	Lower. 95	Upper. 95
MET	0.600	1.822	5.595	2.200E−08	1.476	2.247
ARNTL2	0.569	1.766	5.402	6.580E−08	1.437	2.171
TLE2	− 0.698	0.498	− 5.176	2.270E−07	0.382	0.648
FAM83A	0.202	1.224	5.041	4.630E−07	1.132	1.325
KIF23	0.564	1.758	4.960	7.040E−07	1.407	2.196
ANLN	0.458	1.580	4.919	8.680E−07	1.317	1.896
LAMA3	0.412	1.510	4.877	1.080E−06	1.279	1.781
NUSAP1	0.671	1.957	4.805	1.550E−06	1.488	2.573
TSPOAP1	− 0.400	0.670	− 4.784	1.720E−06	0.569	0.790
ECT2	0.590	1.805	4.775	1.800E−06	1.416	2.300
FAM111B	0.617	1.854	4.763	1.910E−06	1.438	2.390
ANO1	0.520	1.682	4.740	2.130E−06	1.357	2.086
CKAP2L	0.525	1.690	4.739	2.150E−06	1.360	2.100
CEP55	0.517	1.677	4.690	2.730E−06	1.351	2.081
ADGRG6	0.437	1.548	4.686	2.780E−06	1.289	1.858
CA12	0.278	1.321	4.683	2.830E−06	1.176	1.484
DLGAP5	0.507	1.660	4.665	3.090E−06	1.342	2.054
DEPDC1	0.423	1.527	4.614	3.940E−06	1.276	1.828
EREG	0.205	1.227	4.610	4.030E−06	1.125	1.339
MMP28	0.397	1.488	4.601	4.210E−06	1.256	1.762
TPX2	0.457	1.580	4.587	4.500E−06	1.299	1.920
CENPE	0.600	1.822	4.585	4.540E−06	1.410	2.355
INPP4B	0.543	1.722	4.566	4.970E−06	1.363	2.174
GPR87	0.188	1.207	4.566	4.980E−06	1.113	1.309

**Fig. 4:**
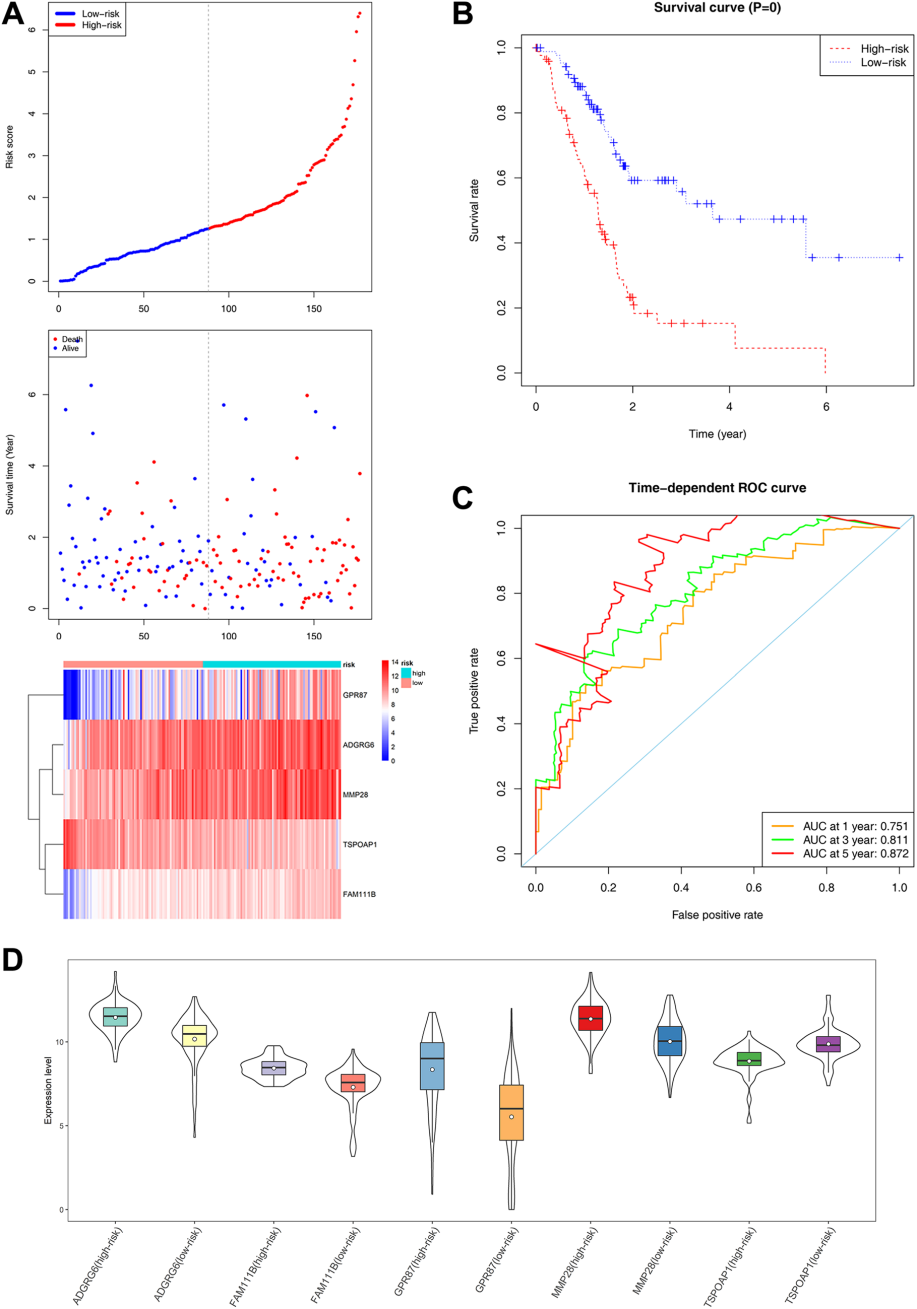
A five-gene prognostic signature for PC. **A** Patient characteristics are ranked according to the risk score. From the top to bottom, they are the risk score of the samples in the high and low risk groups, the distribution of survival status, and the heat map of the five genes. The dotted line indicates the patients in the high and low risk groups divided by the median risk score of 1.273. The left side of the dotted line is low risk, the right side is high risk, and the risk value of patients from left to right increases in turn. **B** Kaplan–Meier survival curve of samples from high and low risk groups. **C** Time-dependent ROC curve for the survival of PC patients predicted by risk score. **D** Expression of the five genes in high- and low-risk groups

**Fig. 5:**
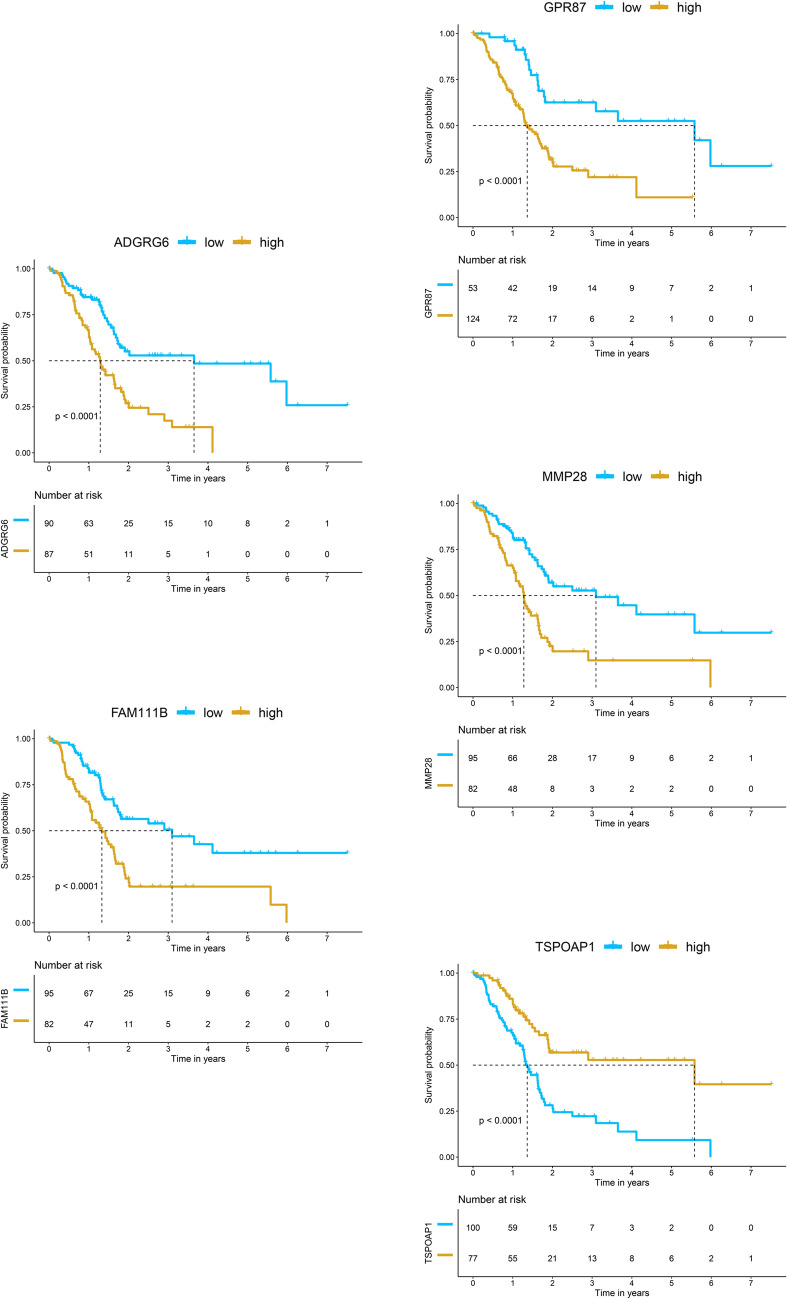
The Kaplan–Meier survival curves for *TSPOAP1, ADGRG6, GPR87, FAM111B* and *MMP28* in the low- and high- risk groups. These 5 genes can significantly differentiate the survival time of PC patients (Log-rank test *P* < 0.0001)

Eighty-eight patients with a risk score higher than the median risk score (1.273) were assigned to the high-risk group, while the remaining 89 patients were assigned to the low-risk group (Fig. 4A). The Kaplan–Meier survival analysis showed a highly significant difference in OS was detected between the high- and low-risk groups (Log-rank test *P* < 0.0001), suggesting that the expression of these 5 genes can be effectively distinguished the high- and low-risk of these PC patients (Fig. 4B and Fig. 5). Similarly, the hub targets of PC had a very strong correlation with the survival time of PC patients (Additional file 1: Fig. S1). The area under the time-dependent ROC curve of the 1-year, 3-year and 5-year survival rates of the patients were 0.751, 0.811 and 0.872, respectively, indicating that the prognostic gene model had good prediction accuracy (Fig. 4C). Figure 4D shows the distribution of the expression levels of the five genes in high- and low-risk groups. Then, we used the GSE15471 (GPL570) dataset from the GEO database as the external validation dataset of WGCNA results. Its pairs of normal and tumor tissue samples were obtained at the time of surgery from resected pancreas of 36 pancreatic cancer patients. Consistent with the WGCNA results, in the GSE15471 dataset, the *TSPOAP1* in the tumor group was significantly lower expressed, while the *ADGRG6, GPR87, FAM111B* and *MMP28* were significantly higher expressed compared with the normal group (Additional file 2: Fig. S2).

### Compound- putative target network

**Fig. 6:**
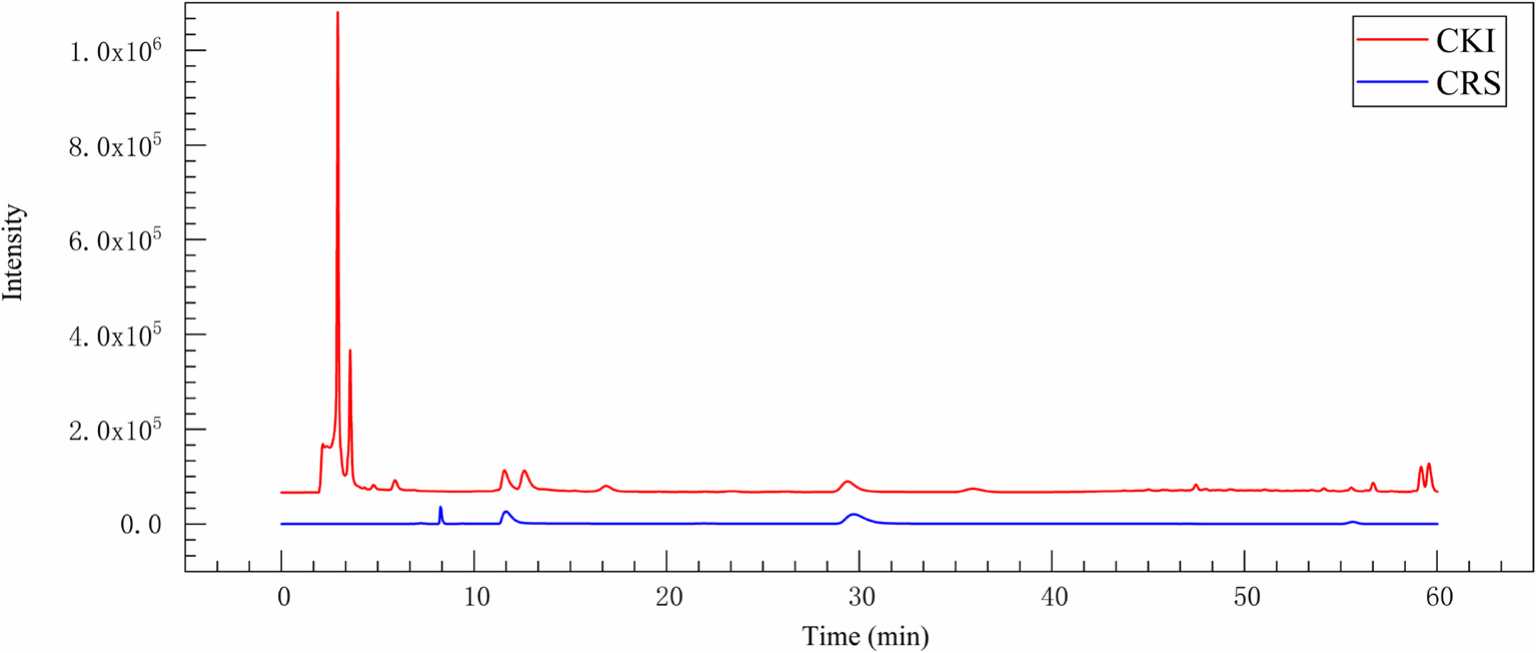
The HPLC–MS of CKI and standard substances. The red and the blue represents CKI and standard substances respectively. 10 compounds were identified by HPLC–MS, including oxysophocarpine, matrine, sophocarpine, sophoridine, oxynamatrine, *N*-methylcytisine, sophoranol, liriodendrin, trifolirhizin and macrozamin

**Fig. 7:**
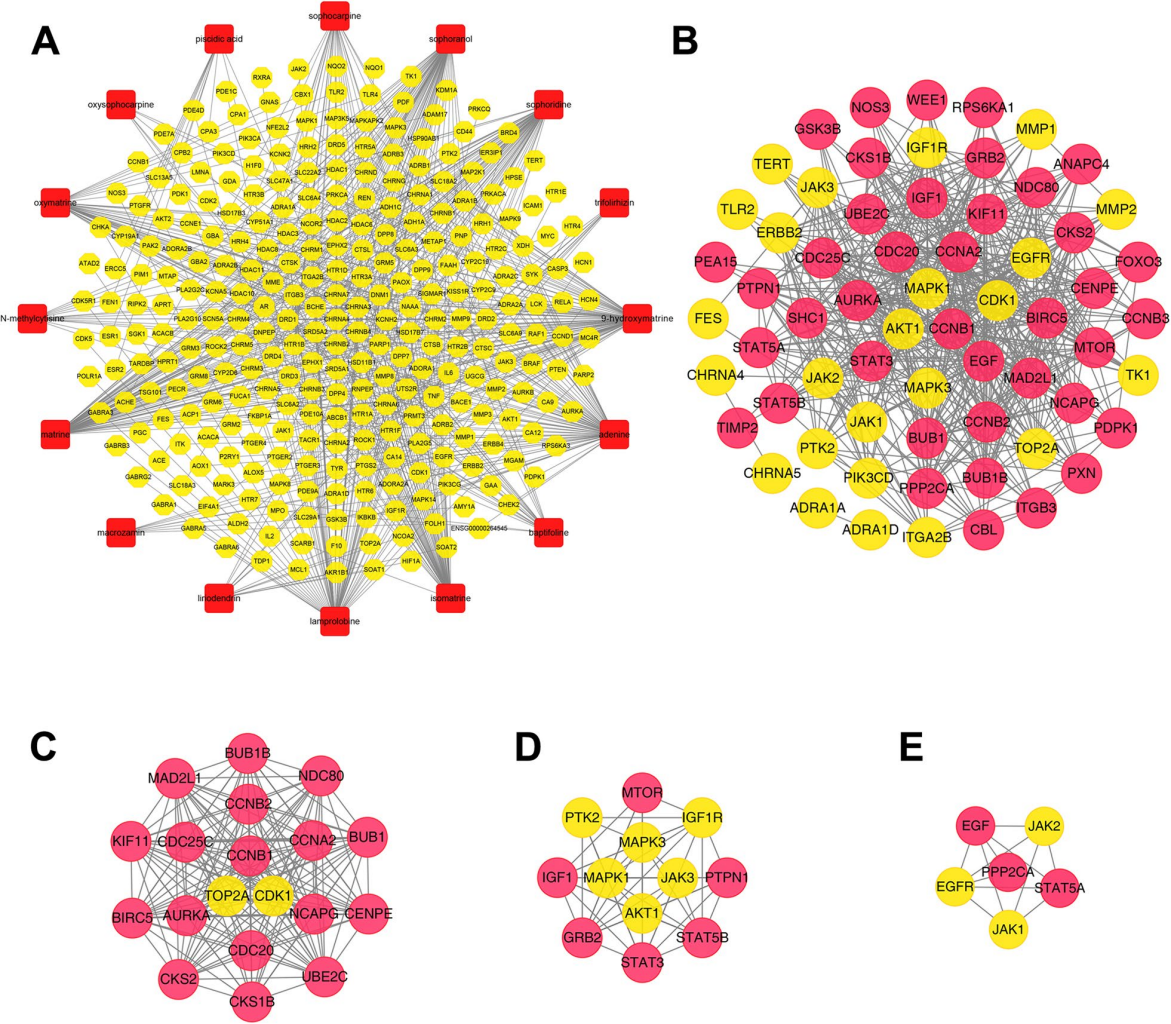
Network analysis related to CKI. **A** Compound-putative target network. The red rectangles represent the compounds of CKI, and the yellow octagons represent corresponding targets. **B** CKI-PC PPI network. **C** Module 1 (score = 18.444). **D** Module 2 (score = 9.273). **E** Module 3 (score = 4.800). The yellow nodes represent the intersections of compound targets and PC targets, and the red nodes represent other human proteins

In our study, 10 marker ingredients of CKI were identified by HPLC–MS (Fig. 6; Table 3). Concurrently, we also supplemented the chemical ingredients by literature research [10, 42]. Collectively, a total of 16 active ingredients in CKI were selected for the next in-depth study, which included 9α-hydroxymatrine, adenine, baptifoline, isomatrine, lamprolobine, piscidic acid and the 10 compounds detected by HPLC–MS. The 16 chemical components of CKI are shown in the Additional file 3: Table S1. The compound- putative target network includes 301 nodes (16 compound nodes and 285 target nodes) and 636 edges (Fig. 7A).

**Table 3:** Major compounds in CKI identified by HPLC–MS

No.	Theoretical value	PPM	Time (min)	MS/MS ions	Structure	Rresumption
1	218.10577	− 4.06	6.007	152.01067, 123.00785, 108.02056, 85.02821, 151.03957		*N*-methylcytisine
2	246.17360	2.94	2.135	163.07516, 134.09622, 59.04988		Sophocarpine
3	248.18896	− 3.67	2.351	179.05530,161.04527,89.02322		Sophoridine
4	248.18910	− 3.17	1.834	176.3145, 148.2457, 112.2569		Matrine
5	262.16841	− 3.65	2.592	181.05049,119.03400,89.02325		Oxysophocarpine
6	264.18394	− 0.52	24.975	247.2548, 205.7581, 148.2567		Oxymatrine
7	264.18395	− 3.19	50.755	211.06097, 167.07062, 149.05995, 123.04412, 71.01385		Sophoranol
8	384.22630	4.13	3.381	110.0603, 96.04457, 94.06555, 92.04989, 67.05498		Macrozamin
9	446.12215	− 2.50	2.976	263.07761,221.06653,179.05548,161.04482		Trifolirhizin
10	764.25163	0.84	17.229	152.01073, 123.04407, 108.0206,		Liriodendrin

### Genes associated with PC

After analysis of the RNA sequencing data, 623 DEGs were identified. In addition, 71 PC-related genes were obtained from the TTD database. Furthermore, 10 hub genes and 5 prognosis-related genes were integrated with the above genes. After deleting duplicate genes, a total of 702 genes were obtained (Additional file 4: Table S2).

### CKI-PC PPI network and core targets identification

PPI networks have been proven to be conducive to explaining complex interactions between multiple proteins in some complex diseases (including cancer) [43]. In order to gain an in-depth understanding of the complex interactions between the intersection targets and further explore the potential mechanism of CKI in the treatment of PC, we construct a CKI-PC PPI network. As shown in Fig. 7A, the network consists of 64 nodes (24 intersection targets of compound targets and PC targets, and 40 other human proteins) and 480 edges. The network analysis showed that the average values of "degree", "betweenness" and "closeness" of the nodes were 15, 0.018331626 and 0.497056362, respectively. In this study, the nodes whose "degree", "betweenness" and " closeness" were all greater than the corresponding average value were selected as key nodes of the network. Finally, a total of 15 potential key targets were obtained, namely AKT1, MAPK1, CCNB1, MAPK3, EGFR STAT3, PPP2CA, CDC25C, EGF, PTPN1, CCNA2, AURKA, BIRC5, CDK1 and JAK1 (Additional file 5: Table S3). The network module is defined as a set of highly interconnected nodes that help to discover and reveal hidden biological information in the network [44]. In this study, a CKI-PC PPI network was divided into 4 modules, and finally, the first 3 modules with scores greater than 4.5 were selected as the key modules (Fig. 7B–E). Among all the 15 potential key targets, 6 targets were clustered in these 3 modules and were directly related to the compound, which were confirmed to be the core targets. The six core targets are RAC-alpha serine/ threonine-protein kinase (AKT1), mitogen-activated protein kinase 1 (MAPK1), Mitogen-activated protein kinase 3 (MAPK3), epidermal growth factor receptor (EGFR), cyclin-dependent kinases 1 (CDK1) and protein tyrosine kinase (janus kinase 1, JAK1).

### GO and KEGG enrichment analysis

**Fig. 8:**
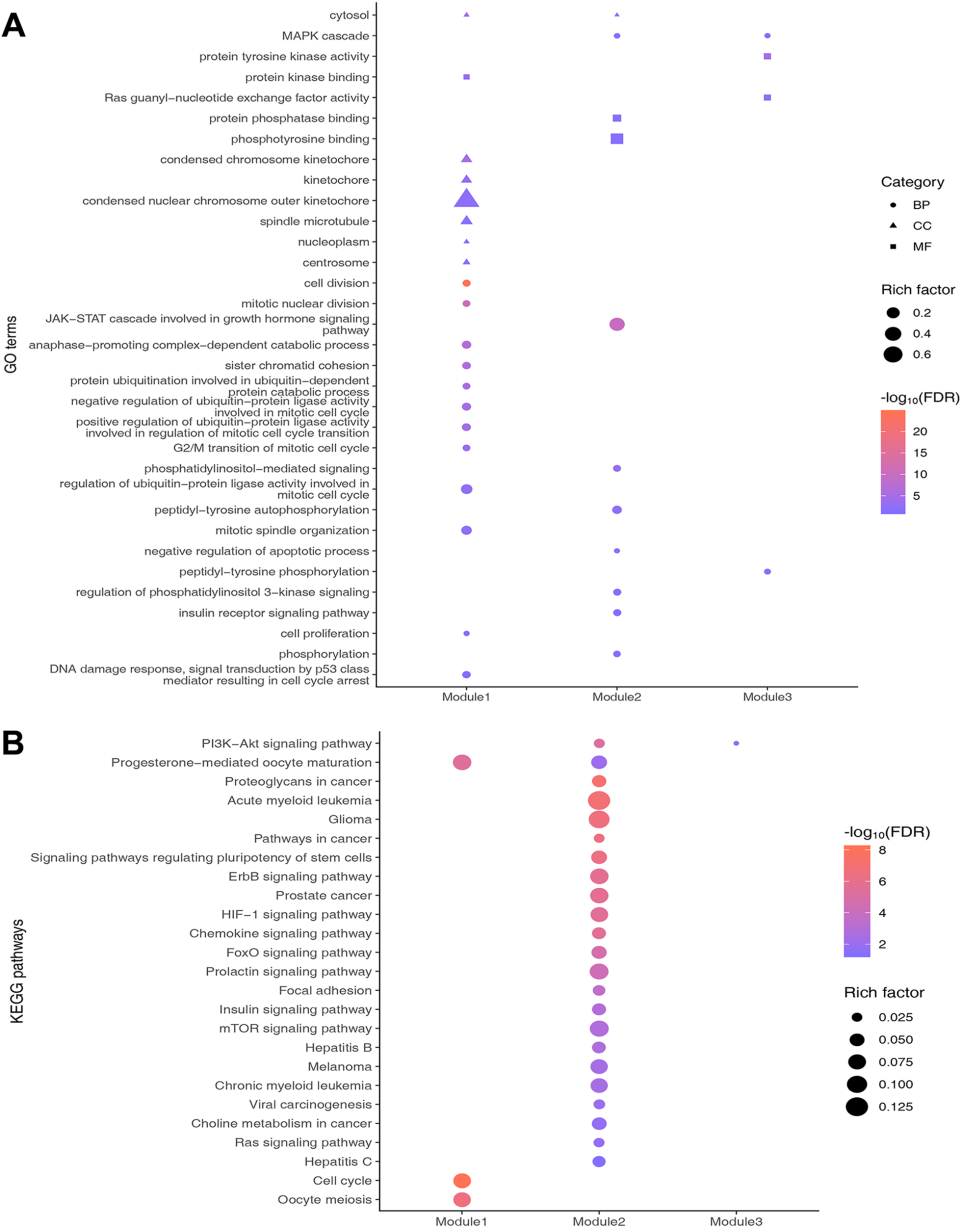
GO and KEGG enrichment analysis of the key modules. **A** GO enrichment analysis. The y-axis represents GO terms, and the x-axis represents three key modules. The circle represents BP, the triangle represents CC, and the rectangle represents MF. **B** KEGG pathway enrichment analysis. The y-axis represents the KEGG pathways, and the x-axis represents the three key modules. Rich factor refers to the ratio of the number of genes in the GO function or KEGG pathway to the number of all the annotated genes enriched in the GO functional or KEGG pathway

**Fig. 9:**
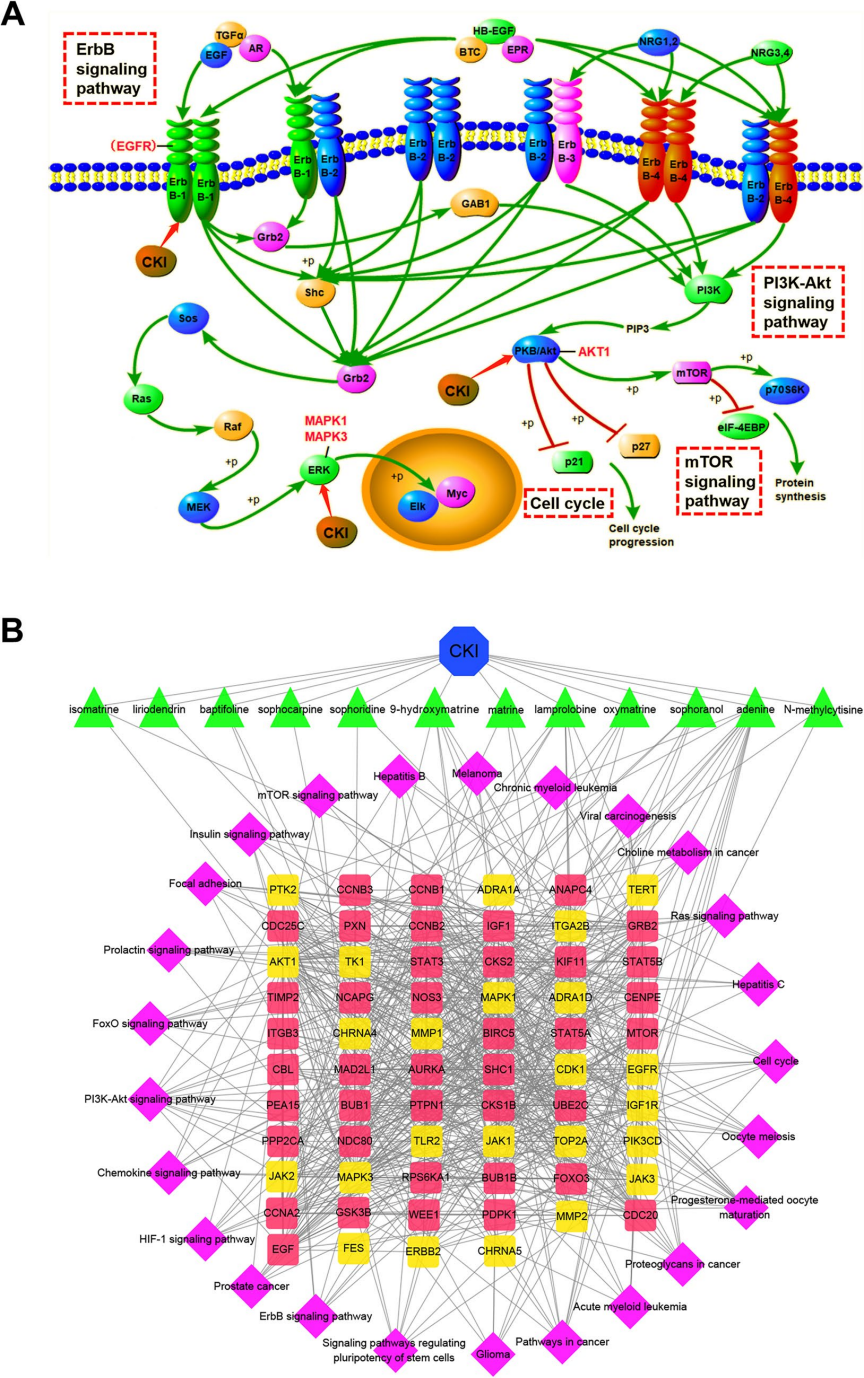
Multipathway mechanism of CKI treating PC. **A** Important pathways in the 3 key modules. The red letters represent the core genes enriched in these pathways. **B** Drug-Compound-PPI Target-Pathway network. Blue octagon represents CKI, green triangles represent compound related to intersection targets, yellow rectangles represent intersection targets of compound targets and PC targets, the red rectangles represent other human proteins, and the purple diamonds represent the relevant pathways enriched by the three modules

In the current study, the biological processes (BP), molecular functions (MF), cellular components (CC) and signalling pathways involved in the three key modules were further explored through GO enrichment and KEGG pathway enrichment analysis. The GO enrichment analysis showed that module 1 was closely related to the cell cycle and cell proliferation; module 2 was closely related to JAK-STAT, MAPK cascade, phosphatidylinositol-mediated signalling, phosphorylation, and negative regulation of the apoptotic process; and module 3 was closely related to protein tyrosine kinase activity and MAPK cascade (Fig. 8A). The KEGG pathway enrichment analysis showed that module 1 was mainly associated with the cell cycle; module 2 was mainly associated with cancer pathway, ErbB signalling pathway, PI3K-Akt signalling pathway, mTOR signalling pathway; and module 3 was associated with PI3K-Akt signalling pathway (Fig. 8B). Figure 9A shows the important pathways in the three modules. To wholly and systematically explain the mechanism of CKI treating PC, Cytoscape software was utilized to construct a Drug-Compound-PPI Target -Pathway network. As shown in Fig. 9B, the network consists of 102 nodes (1 node of CKI, 12 nodes of compounds related to intersection targets, 64 nodes of PPI targets, 25 nodes of pathways enriched by three modules) and 723 edges.

### Molecular docking

**Fig. 10:**
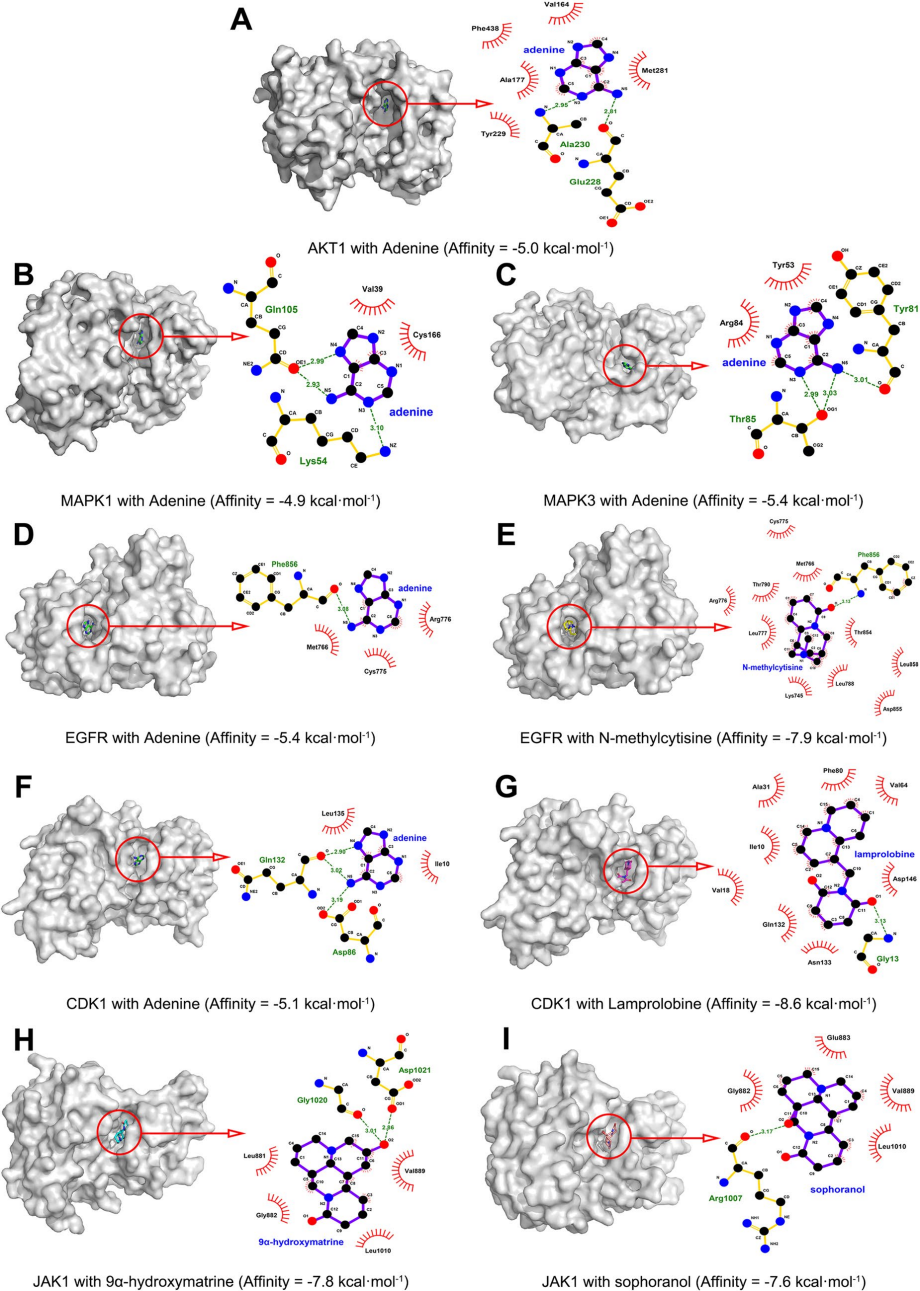
Molecular docking of the core targets with its corresponding compound. Schematic diagram of the combination of **A** AKT1 and adenine; **B** MAPK1 and adenine; **C** MAPK3 and adenine; **D** EGFR and adenine; **E** EGFR and *N*-methylcytisine; **F** CDK1 and adenine; **G** CDK1 and lamprolobine; **H** JAK1 and 9α-hydroxymatrine; **I** JAK1 and sophoranol

To explore the binding methods between core targets and related CKI compounds, the 6 targets, including AKT1, MAPK1, MAPK3, EGFR, CDK1 and JAK1 were used for molecular docking verification. The crystal structures of the 6 targets were retrieved from the PDB database and the 3D structures of the 5 compounds (adenine, *N*-methylcytisine, lamprolobine, 9α-hydroxymatrine and sophoranol) were downloaded from the PubChem database. The docking operation (the docking results are shown in Table 4) was performed by AutoDock Vina. Figure 10 shows the binding methods between compounds and targets. Adenine mainly forms 2 hydrogen bonds with residues Ala230 and Glu228 on AKT1 protein, and a total of 5 residues are bound to the protein by hydrophobic interaction. Adenine mainly forms 3 hydrogen bonds with residues Lys54 and Gln105 on MAPK1 protein, and a total of 2 residues bind to the protein by hydrophobic interaction. Adenine mainly forms 3 hydrogen bonds with residues Tyr81 and Thr85 on MAPK3 protein, and a total of 2 residues bind to the protein by hydrophobic interaction. Adenine mainly forms a hydrogen bond with Phe856 residues on EGFR protein, and a total of 3 residues bind to the protein by hydrophobic interaction. *N*-methylcytisine mainly forms a hydrogen bond with Phe856 residue on EGFR protein, and a total of 10 residues are bound to the protein by hydrophobic interaction. Adenine mainly forms 3 hydrogen bonds with Asp86 and Gln132 residues on CDK1 protein, and a total of 2 residues bind to the protein by hydrophobic interaction. Lamprolobine mainly forms a hydrogen bond with the Gly13 residue on the CDK1 protein, and a total of 8 residues are bound to the protein by hydrophobic interaction. 9α-hydroxymatrine mainly forms 2 hydrogen bonds with Gly1020 and Asp1021 residues on JAK1 protein, and a total of 4 residues bind to the protein by hydrophobic interaction. Sophoranol mainly forms a hydrogen bond with the Arg1007 residue on the JAK1 protein, and a total of 4 residues are bound to the protein by hydrophobic interaction.

**Table 4:** Docking results of 6 core targets and related compounds

Targets	PDB ID	Compound	Affinity (kcal·mol^−1^)
AKT1	6CCY	Adenine	− 5.0
MAPK1	5NHH	Adenine	− 4.9
MAPK3	4QTB	Adenine	− 5.4
EGFR	1XKK	Adenine	− 5.4
		*N*-methylcytisine	− 7.9
CDK1	5LQF	Adenine	− 5.1
		Lamprolobine	− 8.6
JAK1	3EYH	9α-Hydroxymatrine	− 7.8
		Sophoranol	− 7.6

### Anti-PC effect of CKI and core targets verification

**Fig. 11:**
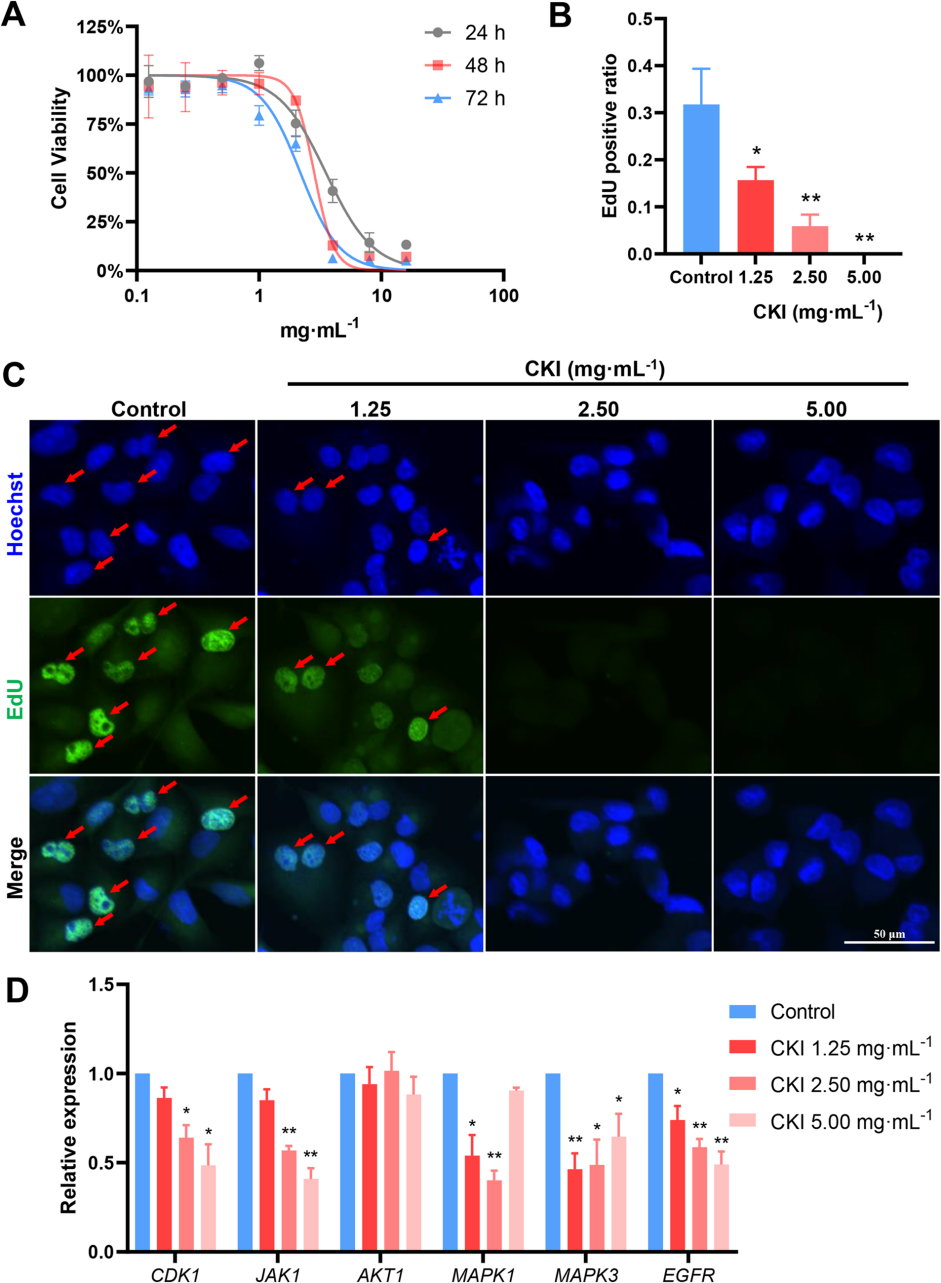
Anti-PC effect of CKI and core targets verification in panc-1 cells. **A** Dose-inhibition curves of CKI at 24 h, 48 h and 72 h. IC_50_ = 3.38 ± 1.40 mg mL^−1^ (24 h); IC_50_ = 2.20 ± 0.54 mg mL^−1^ (48 h); IC_50_ = 1.84 ± 0.38 mg mL^−1^ (72 h). **B**, **C** The proliferation of panc-1 cells was detected by EdU incorporation assay, and observed by confocal microscopy. 10 μM EdU concentration. 2 h labelling time. **D** The relative mRNA expression of core targets was measured by RT-qPCR after CKI intervention. Data were presented as mean ± SD. *n* = 3. *P < 0.05; **P < 0.01

Panc-1 cells were used to observe the anti-proliferation effect of CKI in PC treatment. Figure 11A exhibited that CKI had similar inhibitory proliferation effects at 24 h, 48 h and 72 h incubation time. Specifically, it was found that the half-maximal inhibitory concentration (IC_50_) values of CKI on panc-1 cells at 24 h was found to be 3.38 ± 1.40 mg mL^−1^. And after 48 h and 72 h, the IC_50_ values of CKI were 2.20 ± 0.54 mg mL^−1^ and 1.84 ± 0.38 mg mL^−1^ respectively. Based on the IC_50_ values and cellular state, 48 h incubated time was chosen for the EdU incorporation assay. The EdU incorporation assay showed that (Fig. 11B, C), EdU positive cells were significantly reduced (*P* < 0.05 or *P* < 0.01) in all CKI groups compared with the control group. It confirmed that CKI could restrain the strong proliferation of PC cells.

**Fig. 12:**
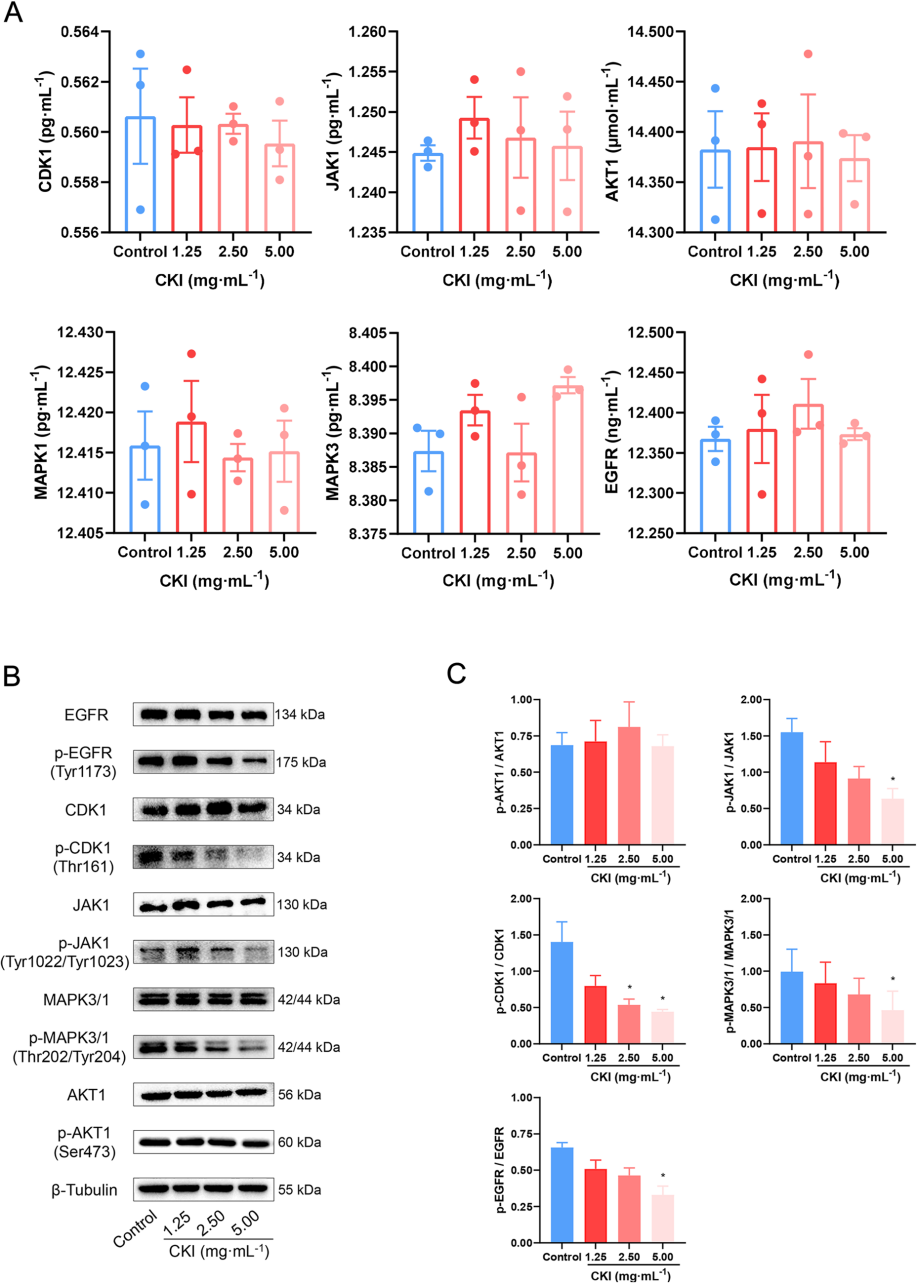
The verification of core targets at protein level with panc-1 cells. **A** AKT1, CDK1, JAK1, EGFR, MAPK1 and MAPK3 protein expression levels were quantified by ELISA after CKI intervention. **B**, **C** p-AKT1, p-CDK1, p-JAK1, p-EGFR, p-MAPK1 and p-MAPK3 protein expression levels were measured by western blot after CKI intervention. Data were presented as mean ± SD. *n* = 3. *P < 0.05

In addition, we also used RT-qPCR assay to analyse the regulatory effects of CKI on core genes (*AKT1, CDK1, JAK1, EGFR, MAPK1* and *MAPK3*) to evaluate the mechanism of CKI in PC treatment. As shown in Fig. 11D, CKI significantly inhibited the expression of *CDK1, JAK1, EGFR, MAPK1* and *MAPK3* (all *P* < 0.05). However, *AKT1* results showed no significant difference in all CKI groups compared with the control group (*P* > 0.05). We followed up with validation experiments on the protein expression levels of the core targets. ELISA results showed that there was no significant difference in total protein content of AKT1, CDK1, JAK1, EGFR, MAPK1 and MAPK3 in the cytoplasm after CKI intervention (all *P* > 0.05, Fig. 12A). Considering that the phosphorylation levels of these proteins were the markers of their own activation or mediated activation of related pathways, we examined the expression levels of their phosphorylated proteins by western blot. Interestingly, after CKI intervention, the expression levels of phosphorylated CDK1, JAK1, EGFR, MAPK1 and MAPK3 were significantly reduced compared with the control cells (all *P* < 0.05, Fig. 12B, C). This was consistent with the results of mRNA expression.

## Discussion

Currently, pancreatic cancer, with high metastasis and a 5-year survival rate of less than 8% of malignant tumours, is responsible for about the same number of deaths due to its poor prognosis [1, 4]. Understanding the pathogenic mechanisms of PC, especially the key survival-related targets associated with clinical information, can be useful in identifying biomarkers for early diagnosis and prognosis [45]. It is reported that *FAM111B* mutation may be associated with PC predisposition based on clinical and molecular evidence [46]. Cancer stem cells are of crucial importance in drug resistance and tumour relapse. Moreover, patients with high *GPR87* showed a poorer prognosis. Further studies assumed that *GPR87* promotes the proliferation of PC stem cells, thus enhancing the malignancy of tumour [47]. Recently, ncRNAs have played a distinctive role in cancer development and immune regulation [48]. As regulatory targets of some ncRNAs, *TSPOAP1, ADGRG6, MMP28* and other genes are particularly important in the initiation, progression of various cancers such as PC. Moreover, their mutations are closely related to immune infiltration and survival rate [49–51]. Therefore, we can speculate that these 5 genes, which may play important roles in PC carcinogenesis, progression and prognosis, could become the PC candidate biomarkers.

Although network pharmacology features high-throughput, systematic and holistic research, it also has many limitations, such as the lack of clinical information [52]. Here, WGCNA analysis is a novel systems biology approach to classify highly co-expressed genes and connect them in the network [17]. And the sub-network regions, are called modules, can be associated with clinical parameters such as cancer status or patient survival [53]. Within our study, the WGCNA results showed that the black and blue modules had the highest correlation with tumour grade and tumour stage, respectively, and both were also strongly associated with patient survival status. Further multivariate Cox proportional hazards regression analysis revealed that among the 5 survival time-related genes, *TSPOAP1* was the protective gene; in contrast, *ADGRG6, GPR87, FAM111B* and *MMP28* were the risk genes. Remarkably, GPR87-mediated activation of the JAK/STAT signalling pathway promoted the expansion of PC stem cells, contributing to the chemotherapy resistance of PC cells [47]. To extend, GPR87 trans-activated EGFR to promote scattering and extension of tumour cells [17]. Based on the analysis of tumour and para-tumour tissues of clinical samples with RNA sequencing, it was found that *GPR87* and *MAPKs* were significantly differentially expressed genes [54]. Similarly, a comprehensive whole-genome, transcriptome and clinical dataset called the POG570 cohort, had revealed that alterations in *EGFR, ADGRG6,* and other genes were involved in tumour drug resistance and sensitivity, as well as recurrent noncoding events [50]. ADGRG6 can also form carcinogenic fusion variants with ROS1 to promote the development of EGFR-tyrosine kinase inhibitor resistance in cancer patients with EGFR mutation [55]. ADGRG6 also promoted tumour-stromal angiogenesis and hypoxia-induced retinal angiogenesis through the GATA2/STAT5/ VEGF/MAPKs signalling pathway. More than that, activated VEGF can further activate STAT5 through phosphorylated JAK2, enhancing the STAT5/VEGF/MAPKs signalling pathway response [56]. Conversely, knockdown of *ADGRG6* results in low expression of *HDAC2* and *GLI2*, and inhibits proliferation and arrests cell cycle in cancer cells [57]. On the likeness, FAM111B, as a degradative enzyme, degrades p16 so that could regulate cyclin D1-CDK4-dependent cell cycle progression leading to poor outcomes [58]. Moreover, the high expression of *CDK1* and *MMP28* are jointly regulated by the upstream *RACGAP1*, which is involved in the pathogenesis, cell cycle progression, migration and invasion of PC cells [49]. MAPK signalling pathway activated by nicotinic cholinergic receptors (NCRs) can enhance tumour cell proliferation, accompanied by increased expression of *MMP2* and *MMP28* genes [59]. Therefore, these genes (*CDKs, JAKs, STATs, MAPKs* and *EGFR*) deserve further exploration in PC pathogenesis in addition to the 5 survival-dependent genes described.

Interestingly, in this study, the 5 core genes *CDK1, JAK1, EGFR, MAPK1,* and *MAPK3* targeted by CKI were investigated by WGCNA and network pharmacology analysis and then verified by in vitro experiments. MAPKs, a class of serine/threonine protein kinases that has four different types (ERKs, P38, JNK, and ERK5) in mammals [60]. MAPK1 is significantly upregulated in multiple types of cancer. Overexpression of MAPK1 induces EMT and is associated with tumour cell proliferation, apoptosis, invasion, and metastasis [61–63]. MAPK1 has been proved to be strongly associated with invasion of pancreatic ductal adenocarcinoma cells [64]. In addition, a bioinformatics study showed that MAPK1 is overexpressed in PC and is associated with poor prognosis in PC patients [65]. Aberrant expression of MAPK3 is related to invasion, metastasis and drug resistance of various tumour cells [66]. Bioinformatics studies revealed that MAPK3 is a core gene associated with PC [67]. In addition, it has been reported that activated MAPK3/1 (ERK1/2) overexpression in PC, and the activity of ERK1/2 can protect PC cells from chemotherapy-induced apoptosis [68]. Other studies have shown that phosphorylation of ERK1/2 promotes proliferation, migration and invasion of pancreatic ductal adenocarcinoma cells [69]. Fortunately, matrine and oxymatrine, both of which are the main ingredients of CKI, have been shown to inhibit the phosphorylation of ERK1/2 [70, 71]. EGFR (ErbB1) belongs to the epidermal growth factor receptor (ErbB) family and is widely distributed on the cell surface of mammalian epithelial cells, glial cells and fibroblasts [72]. ErbB2, ErbB3 and ErbB4 also belongs to the ErbB family. When combined with the ligands AR, TGF, and EGF, the combined products activate downstream genes such as *MAPKs* to regulate cell survival, proliferation, differentiation and migration [73]. EGFR is highly expressed in a variety of tumours, and is associated with tumour occurrence and development and poor prognosis [74, 75]. It has been reported that EGFR overexpression can be detected in up to 90% of PC tumour tissues, and that overexpressed EGFR is closely involved in the progression of PC and the poor prognosis of PC patients [76, 77]. It was also confirmed that EGFR is an effective target for PC prevention and treatment [78]. In addition, oxymatrine was found to effectively inhibits EGFR phosphorylation and EGFR-related signalling pathways, thereby inhibiting gastric cell proliferation and invasion [79]. Not only that, matrine and oxymatrine are EGFR-targeted components and can act on EGFR similarly as a control drug gefitinib [80]. CDK1 is closely related to the cell cycle [81]. At the same time, overexpressed CDK1 is associated with PC development and poor prognosis of patients [82]. Besides, CKI has been proven to have an inhibitory effect on CDK1 [83]. Matrine arrests the cell cycle and induces apoptosis in several cancer cell lines, and the mechanism is downregulated cell cycle-related proteins CDK1, Cyclin B1 and Cyclin D1 [84]. Oxymatrine has a similar mechanism as described above to arrest the cell cycle in glioblastoma cells. In detail, it inhibits phosphorylation of EGFR and STAT3, thereby inhibiting the expression of downstream cell cycle-related proteins (CDK1, CDK4 and CDK6) [85]. Coincidentally, it has been reported that Oxymatrine can simultaneously inhibit both EGFR/MAPK3/1 and EGFR/CDK1 pathways to arrest the cycle in multiple cancer cell lines [86]. Matrine, Oxymatrine, and Sophoridine can exert biological effects on multiple proteins in the cell cycle pathway of colorectal cancer cells, thereby arresting cell cycle pathway activation in a full range [87]. JAK is a non-transmembrane tyrosine kinase that can activate the STAT protein under the action of growth factors and cytokines [88]. JAK/STAT signalling pathway is continuously activated in tumour cells, and its inhibition would induce apoptosis of PC cells and their proliferation inhibition [89]. JAK1 is a member of the JAK protein family, which is closely related to the progression of various cancers [90]. Ruxolitinib, a JAK1/JAK2 inhibitor, can inhibit endothelial cell-mediated proliferation of PC cells [91]. It can also treat patients with pancreatic ductal adenocarcinoma who have the characteristics of angiogenesis genes by targeting *JAK1* and *TGF-β* [92]. Matrine can also inhibit cell growth by inhibiting activation of the JAK1/STAT3 signalling pathway [93]. Oxymatrine plays a significant role in regulating cell proliferation and survival in tumor cells by inhibiting the activation of JAK1 and JAK2, thereby inhibiting the phosphorylation and nuclear translocation of STAT5 [94]. Consistent with these reports, our results illustrated that CKI can inhibit the proliferation of pancreatic cancer cells, arrest cell cycle and downregulate the expression levels of p-CDK1, p-JAK1, p-EGFR and pMAPK3/1.

Taken together, we can speculate that the molecular mechanisms of CKI in PC treatment are as follows: (1) CKI inhibits proliferation of PC cells via inhibition of the EGFR transactivation, and downregulation of the NCR-mediated MAPK pathway; (2) CKI inhibits expansion of PC stem cells via inactivation of the GPR87/JAK/STAT pathway; (3) CKI arrests cell cycle progression of PC cells via inhibition of the FAM111B/p16/CDK pathway; (4) CKI inhibits migration and invasion of PC cells via downregulation of the NCR-mediated MAPK/MMP pathway; (5) CKI reverses chemoresistance of PC cells via inhibition of the ROS1-ADGRG6/EGFR pathway; (6) CKI inhibits tumour angiogenesis of PC via inhibition of the ADGRG6/GATA2/STAT5/VEGF/MAPK pathway. However, these speculations should be supported by further basic and clinical experimental data.

## Conclusions

In summary, this study demonstrated a strategy to optimize conventional network pharmacology, and then explained that the molecular mechanism of CKI treating PC was closely associated with 5 core genes (including *CDK1, JAK1, EGFR, MAPK1* and *MAPK3*), which were related to important signalling pathways (including cell cycle, JAK/STAT and ErbB pathway) and survival-correlated genes (including *TSPOAP1, ADGRG6, GPR87, FAM111B* and *MMP28*). These findings may be useful for clinical decision-making and guidance for rational clinical use of CKI in PC treatment.

## Supplementary Information

**Additional file 1: Figure S1.** The Kaplan–Meier survival curves for 10 hub targets.

**Additional file 2: Figure S2.** The external validation for the results of WGCNA.

**Additional file 3: Table S1.** Information about the 16 compounds of CKI.

**Additional file 4: Table S2.** Genes associated with PC.

**Additional file 5: Table S3.** Information of potential key targets in CKI-PC PPI network.

## Data Availability

The data used to support the current study are available from the corresponding author on reasonable request.

## Abbreviations

CKI: Compound kushen injection
PC: Pancreatic cancer
WGCNA: Weighted gene co-expression network analysis
TCM: Traditional Chinese medicine
TCGA: The cancer genome atlas
TOM: Topological overlap measure
ME: Module eigengene
MM: Module membership
GS: Gene significance
MCC: Maximal clique centrality
HR: Hazard ratio
CI: Confidence interval
ROC: Receiver operating characteristic
AUC: Area under curve
TCMSP: Traditional Chinese medicine systems pharmacology database and analysis platform
STITCH: Search tool for interactions of chemicals
STRING: Search tool for the retrieval of interacting genes/proteins
PPI: Protein–protein interaction
MCODE: Molecular complex detection
GO: Gene ontology
KEGG: Kyoto encyclopedia of genes and genomes
SD: Standard deviation
PDB: Protein data bank
TTD: Therapeutic target database
CDK1: Cyclin-dependent kinase 1
MAPK1: Mitogen-activated protein kinase 1
MAPK3: Mitogen-activated protein kinase 3
EGFR: Epidermal growth factor receptor
JAK1: Janus kinase 1
