# Supplementary figures and images for: A network pharmacology approach to reveal the pharmacological targets and biological mechanism of compound kushen injection for treating pancreatic cancer based on WGCNA and in vitro experiment validation
Source: Chin Med. 2021 Nov 22;16:121. doi: 10.1186/s13020-021-00534-y (PMC8607619; doi:10.1186/s13020-021-00534-y)

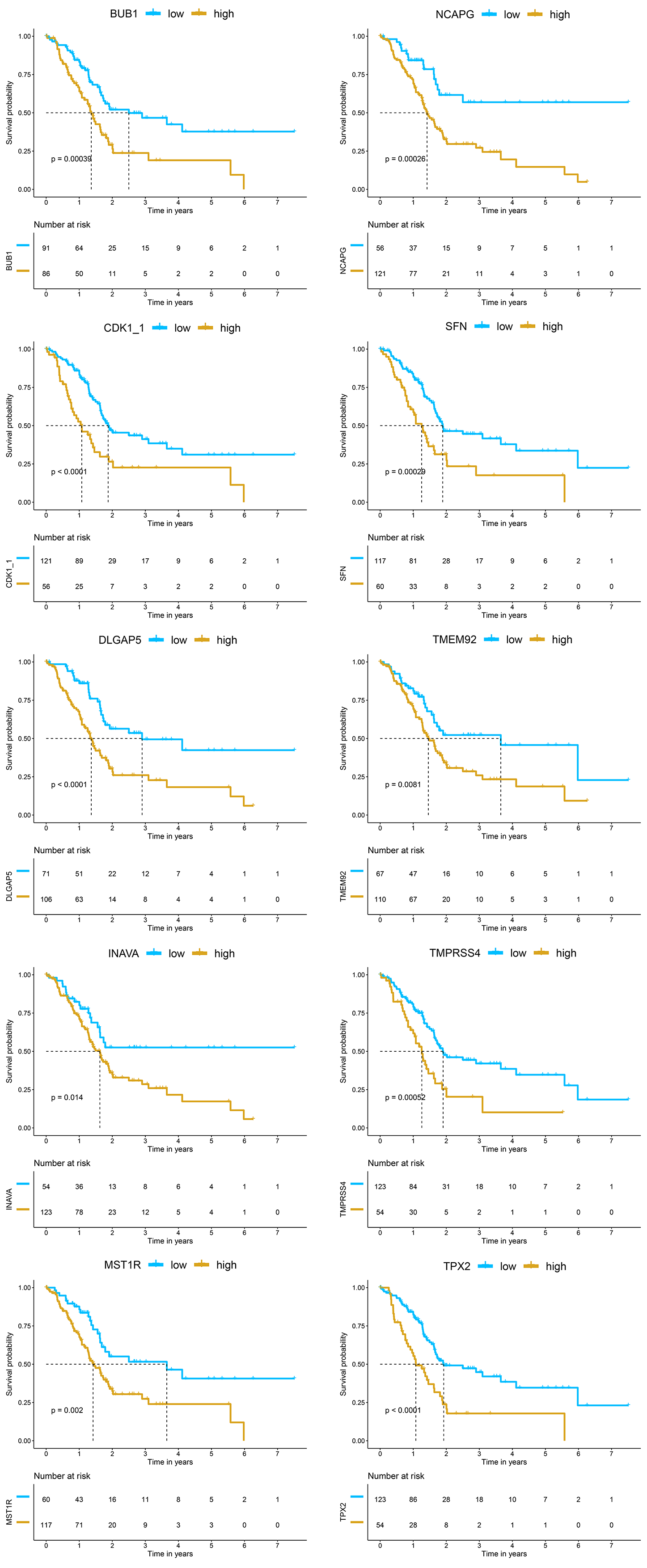

Supplement: Supplementary file 1 — Additional file 1: Figure S1. The Kaplan–Meier survival curves for 10 hub targets. [file 13020_2021_534_MOESM1_ESM.tif]

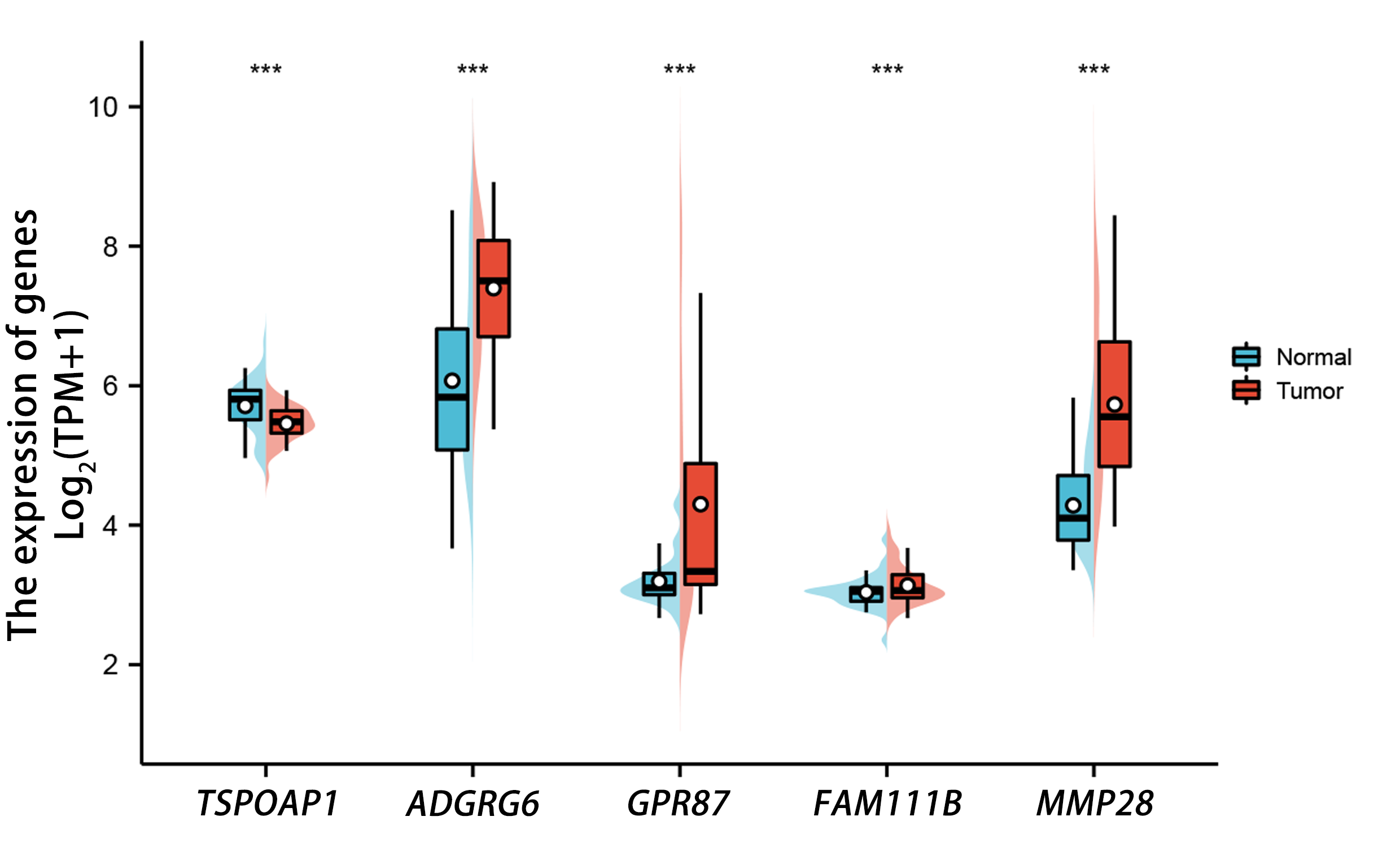

Supplement: Supplementary file 2 — Additional file 2: Figure S2. The external validation for the results of WGCNA. [file 13020_2021_534_MOESM2_ESM.tif]
