# Supplementary material for: A network pharmacology approach to reveal the pharmacological targets and biological mechanism of compound kushen injection for treating pancreatic cancer based on WGCNA and in vitro experiment validation
Source: Chin Med. 2021 Nov 22;16:121. doi: 10.1186/s13020-021-00534-y (PMC8607619; doi:10.1186/s13020-021-00534-y)
Supplement: Supplementary file 3 — Additional file 3: Table S1. Information about the 16 compounds of CKI. [file 13020_2021_534_MOESM3_ESM.docx]

**Table S1.** Information about the 16 compounds of CKI.

| **Compound** | **Pubchem CID** | **MW (g/mol)** | **Structure** |
| --- | --- | --- | --- |
| 9α-hydroxymatrine | 15385684 | 264.369 | 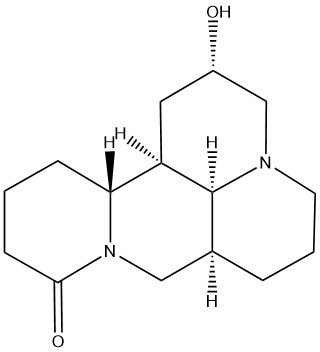 |
| adenine | 190 | 135.13 | 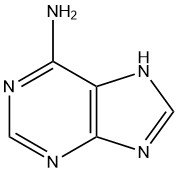 |
| baptifoline | 621307 | 260.337 | 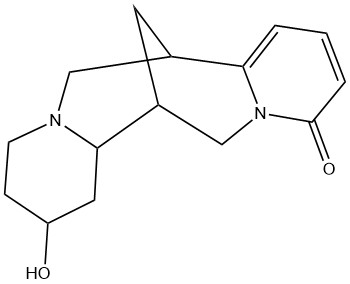 |
| isomatrine | 5271984 | 248.37 | 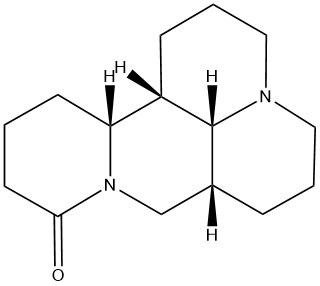 |
| lamprolobine | 87752 | 264.369 | 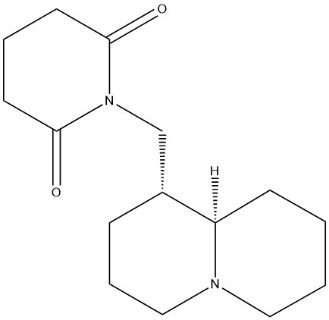 |
| liriodendrin | 21603207 | 742.724 | 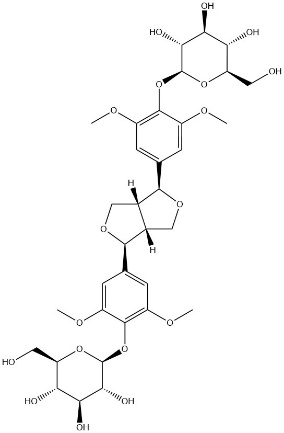 |
| macrozamin | 9576780 | 384.338 | 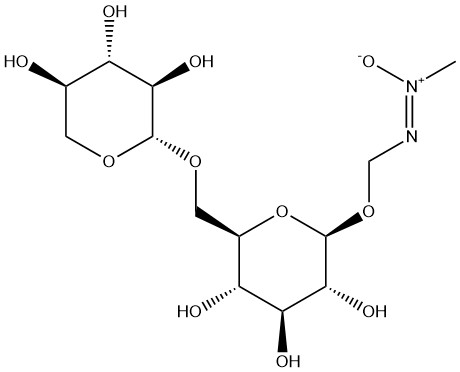 |
| matrine | 91466 | 248.37 | 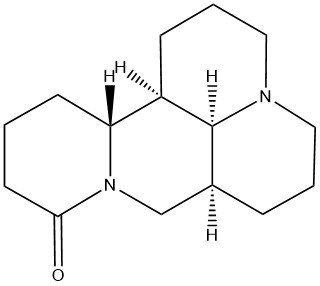 |
| N-methylcytisine | 670971 | 204.273 | 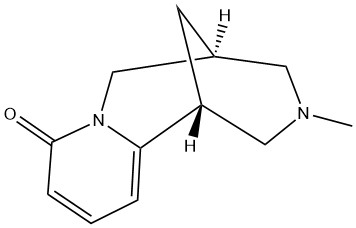 |
| oxymatrine | 114850 | 264.369 | 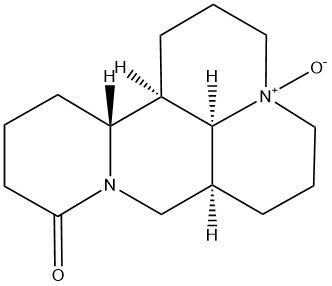 |
| oxysophocarpine | 24721085 | 262.353 | 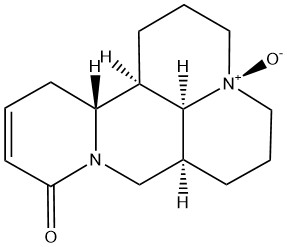 |
| piscidic acid | 6710641 | 256.21 | 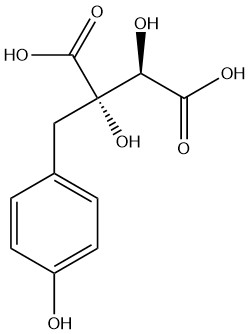 |
| sophocarpine | 115269 | 246.354 | 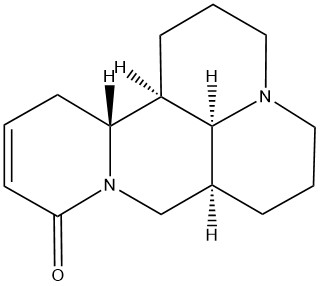 |
| sophoranol | 12442899 | 264.369 | 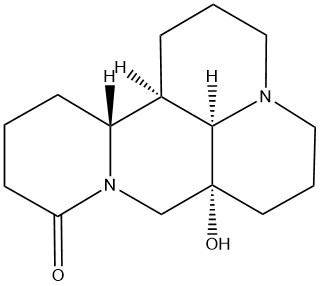 |
| sophoridine | 165549 | 248.37 | 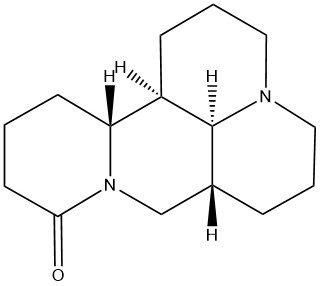 |
| trifolirhizin | 442827 | 446.408 | 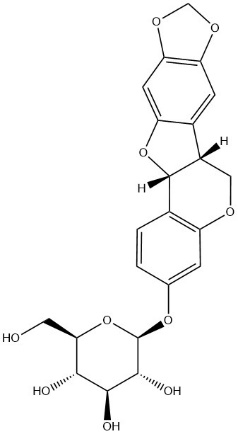 |
