# Supplementary material for: A network pharmacology approach to reveal the pharmacological targets and biological mechanism of compound kushen injection for treating pancreatic cancer based on WGCNA and in vitro experiment validation
Source: Chin Med. 2021 Nov 22;16:121. doi: 10.1186/s13020-021-00534-y (PMC8607619; doi:10.1186/s13020-021-00534-y)
Supplement: Supplementary file 4 — Additional file 4: Table S2. Genes associated with PC. [file 13020_2021_534_MOESM4_ESM.docx]

**Table S2.** Genes associated with PC.

| Gene | Source | Gene | Source | Gene | Source |
| --- | --- | --- | --- | --- | --- |
| NCAPG | WGCNA | RASSF4 | TCGA | JAK3 | TCGA |
| BUB1 | WGCNA | LIPA | TCGA | PLXNA2 | TCGA |
| CDK1 | WGCNA | DOCK8 | TCGA | SERPINA9 | TCGA |
| TPX2 | WGCNA | GDPD5 | TCGA | IL24 | TCGA |
| DLGAP5 | WGCNA | P2RY13 | TCGA | CD79A | TCGA |
| INAVA | WGCNA | HVCN1 | TCGA | PADI4 | TCGA |
| MST1R | WGCNA | HBA1 | TCGA | BEND4 | TCGA |
| TMPRSS4 | WGCNA | NLRC3 | TCGA | IL10RA | TCGA |
| TMEM92 | WGCNA | ABI3 | TCGA | VASH2 | TCGA |
| SFN | WGCNA | SHANK3 | TCGA | PALD1 | TCGA |
| TSPOAP1 | WGCNA | MYO1F | TCGA | CD8A | TCGA |
| ADGRG6 | WGCNA | BLK | TCGA | INPP5D | TCGA |
| GPR87 | WGCNA | TPO | TCGA | DNAH8 | TCGA |
| FAM111B | WGCNA | SP140 | TCGA | FES | TCGA |
| MMP28 | WGCNA | RNF166 | TCGA | LY9 | TCGA |
| CHEK1 | TTD | TBC1D10C | TCGA | TDO2 | TCGA |
| EPCAM | TTD | CEACAM21 | TCGA | TNFAIP8 | TCGA |
| ERBB2 | TTD | ZNF589 | TCGA | TAGAP | TCGA |
| PDGFRA | TTD | CCL2 | TCGA | TREML1 | TCGA |
| IL15RA | TTD | SEMA3D | TCGA | CR2 | TCGA |
| OGDH | TTD | GPR18 | TCGA | C1orf162 | TCGA |
| STAT3 | TTD | GPR55 | TCGA | SMAP2 | TCGA |
| SRC | TTD | GRAP | TCGA | LRMP | TCGA |
| HTR2A | TTD | AKNA | TCGA | GIMAP1 | TCGA |
| CCN2 | TTD | EPB42 | TCGA | MEF2C | TCGA |
| DRD2 | TTD | KIF17 | TCGA | SBK1 | TCGA |
| PROM1 | TTD | GNLY | TCGA | TLR7 | TCGA |
| JAK2 | TTD | FAM129C | TCGA | DENND1C | TCGA |
| JAK1 | TTD | RIPOR2 | TCGA | OR2I1P | TCGA |
| PLAUR | TTD | ITGAX | TCGA | B3GNT3 | TCGA |
| RXRG | TTD | RASGRP2 | TCGA | SELL | TCGA |
| HGF | TTD | ARHGAP9 | TCGA | CMBL | TCGA |
| PTK2 | TTD | GAPT | TCGA | NRROS | TCGA |
| NRAS | TTD | KAZN | TCGA | SIT1 | TCGA |
| MTOR | TTD | GIMAP5 | TCGA | PSTPIP1 | TCGA |
| DNMT1 | TTD | NCF1 | TCGA | CRTAC1 | TCGA |
| AKT1 | TTD | STAP1 | TCGA | IFFO1 | TCGA |
| CSF2 | TTD | SPATC1 | TCGA | BAALC | TCGA |
| CD70 | TTD | TREML2 | TCGA | LAIR1 | TCGA |
| NTRK1 | TTD | GAB3 | TCGA | APOBR | TCGA |
| F3 | TTD | FCRLA | TCGA | ACSM5 | TCGA |
| NOTCH3 | TTD | NPY1R | TCGA | RAC2 | TCGA |
| NOTCH2 | TTD | RUBCNL | TCGA | SIGLEC9 | TCGA |
| MMP1 | TTD | IL12RB1 | TCGA | MYBL1 | TCGA |
| MMP2 | TTD | CD37 | TCGA | TLR1 | TCGA |
| MMP7 | TTD | CD33 | TCGA | EMILIN2 | TCGA |
| MRM2 | TTD | SNX10 | TCGA | ZNF80 | TCGA |
| IGF1R | TTD | CD53 | TCGA | ARL11 | TCGA |
| ERBB3 | TTD | MYO1G | TCGA | STX11 | TCGA |
| ATP1A1 | TTD | CALHM6 | TCGA | SNX20 | TCGA |
| ATP1A2 | TTD | CARMIL2 | TCGA | HTRA4 | TCGA |
| ATP1A3 | TTD | NCKAP1L | TCGA | SSC4D | TCGA |
| ATP1B1 | TTD | IL4I1 | TCGA | GRK3 | TCGA |
| ATP1B2 | TTD | HEATR9 | TCGA | ITGB2 | TCGA |
| ATP1B3 | TTD | ARHGAP30 | TCGA | CYBB | TCGA |
| LTB4R | TTD | FADS3 | TCGA | LILRA2 | TCGA |
| TOP2A | TTD | PSTPIP2 | TCGA | PTPRO | TCGA |
| TOP2B | TTD | PIK3CD | TCGA | LYL1 | TCGA |
| TLR2 | TTD | WAS | TCGA | C1QB | TCGA |
| CSF2RA | TTD | CCR6 | TCGA | NLRP4 | TCGA |
| OGFR | TTD | LIMD2 | TCGA | HLA-DQA2 | TCGA |
| FLT1 | TTD | TRAF3IP3 | TCGA | RENBP | TCGA |
| TERT | TTD | NOX5 | TCGA | ABCA12 | TCGA |
| TK1 | TTD | HLA-DOB | TCGA | ASTL | TCGA |
| EGFR | TTD | PITPNM3 | TCGA | LIPE | TCGA |
| RRM2 | TTD | RALGPS2 | TCGA | CLEC2D | TCGA |
| CEACAM5 | TTD | HCK | TCGA | SUSD3 | TCGA |
| KRAS | TTD | AMTN | TCGA | VMO1 | TCGA |
| MSLN | TTD | ANKDD1A | TCGA | OLR1 | TCGA |
| MUC1 | TTD | KLF1 | TCGA | SH2D3C | TCGA |
| CLDN18 | TTD | SEPT1 | TCGA | CDH23 | TCGA |
| TGFBR1 | TTD | KRT75 | TCGA | FRMD4A | TCGA |
| TGFB1 | TTD | NLRC5 | TCGA | ARHGAP45 | TCGA |
| IDO1 | TTD | ZAP70 | TCGA | TSPAN1 | TCGA |
| TP53 | TTD | TTC24 | TCGA | SOD2 | TCGA |
| CXCR4 | TTD | AC119396.1 | TCGA | FXYD3 | TCGA |
| MAPK3 | TTD | SIGLEC5 | TCGA | CCL4L2 | TCGA |
| MAPK1 | TTD | GYPE | TCGA | SYNE3 | TCGA |
| EPHB2 | TTD | GLYATL1B | TCGA | TP53INP1 | TCGA |
| CD5L | TCGA | BACH2 | TCGA | PIK3AP1 | TCGA |
| LMAN1L | TCGA | CX3CR1 | TCGA | CAMK1D | TCGA |
| FAM9C | TCGA | BCL11A | TCGA | AL133352.1 | TCGA |
| SPIC | TCGA | LILRB2 | TCGA | SLC7A10 | TCGA |
| KCNT1 | TCGA | LPXN | TCGA | CD84 | TCGA |
| STAB2 | TCGA | INTS6L | TCGA | SH2D1B | TCGA |
| CD160 | TCGA | C10orf90 | TCGA | CCDC88B | TCGA |
| NKX2-5 | TCGA | RASAL3 | TCGA | IL2RB | TCGA |
| SLC9A5 | TCGA | FAM78A | TCGA | RASGRP1 | TCGA |
| GPR182 | TCGA | ADAP2 | TCGA | CR1L | TCGA |
| ITGAD | TCGA | SLC12A3 | TCGA | GCSAM | TCGA |
| MMP12 | TCGA | SCN4A | TCGA | TMEM110-MUSTN1 | TCGA |
| AC126755.2 | TCGA | LCP1 | TCGA | RNF144B | TCGA |
| KLRF1 | TCGA | CHODL | TCGA | CERKL | TCGA |
| SCN11A | TCGA | PDE7A | TCGA | TOB1 | TCGA |
| ADRA1A | TCGA | AC068896.1 | TCGA | ARHGAP15 | TCGA |
| DNASE1L3 | TCGA | FABP4 | TCGA | PPP1R14D | TCGA |
| GDF7 | TCGA | DOCK2 | TCGA | VPREB3 | TCGA |
| NR5A1 | TCGA | FLI1 | TCGA | DOK2 | TCGA |
| KLRD1 | TCGA | SLC4A1 | TCGA | SPATA21 | TCGA |
| PARP15 | TCGA | CHI3L2 | TCGA | RELT | TCGA |
| FCRL3 | TCGA | RCSD1 | TCGA | C1QC | TCGA |
| H3F3C | TCGA | FGD3 | TCGA | SFMBT2 | TCGA |
| NCR1 | TCGA | TRAF1 | TCGA | TTN | TCGA |
| CCM2L | TCGA | CTSW | TCGA | C19orf38 | TCGA |
| SIGLEC11 | TCGA | GNGT2 | TCGA | STYK1 | TCGA |
| AIF1L | TCGA | TFEC | TCGA | NCF2 | TCGA |
| FGFBP2 | TCGA | NME8 | TCGA | GPR174 | TCGA |
| CR1 | TCGA | NCR3 | TCGA | LCP2 | TCGA |
| CD22 | TCGA | EVI2B | TCGA | HLA-DOA | TCGA |
| KLHL14 | TCGA | FCMR | TCGA | ARHGDIB | TCGA |
| TBX21 | TCGA | EVL | TCGA | KLF12 | TCGA |
| CRHBP | TCGA | PDE6G | TCGA | DPEP3 | TCGA |
| TRAPPC3L | TCGA | LTB | TCGA | GFRA2 | TCGA |
| LEFTY2 | TCGA | KIR3DL1 | TCGA | PREX1 | TCGA |
| FCRL2 | TCGA | IRX6 | TCGA | GRAPL | TCGA |
| CLECL1 | TCGA | FAM49A | TCGA | PGLYRP1 | TCGA |
| LILRA1 | TCGA | BTNL9 | TCGA | SLFN14 | TCGA |
| TLR10 | TCGA | BCL2 | TCGA | APOE | TCGA |
| ATP4B | TCGA | DPEP2 | TCGA | CBLN3 | TCGA |
| FCRL5 | TCGA | KIR2DL3 | TCGA | MYBPC3 | TCGA |
| FCRL6 | TCGA | FAM178B | TCGA | PRAM1 | TCGA |
| CD36 | TCGA | PLCB2 | TCGA | P2RY12 | TCGA |
| PTGDR | TCGA | CELF2 | TCGA | ZIC2 | TCGA |
| CHRNA4 | TCGA | CD226 | TCGA | ALS2CR12 | TCGA |
| P2RX5 | TCGA | RHOH | TCGA | POU2AF1 | TCGA |
| BFSP2 | TCGA | SNX22 | TCGA | APOC1 | TCGA |
| FGD2 | TCGA | PF4V1 | TCGA | TNFSF8 | TCGA |
| HMOX1 | TCGA | OR52N4 | TCGA | JPH1 | TCGA |
| CFP | TCGA | CXCR5 | TCGA | GZMH | TCGA |
| C11orf21 | TCGA | STAB1 | TCGA | ZNF578 | TCGA |
| ATG16L2 | TCGA | PIK3R6 | TCGA | ADRA1D | TCGA |
| IL34 | TCGA | SPI1 | TCGA | NUGGC | TCGA |
| DOK3 | TCGA | RAB42 | TCGA | SLC7A11 | TCGA |
| KLRC4-KLRK1 | TCGA | CCL14 | TCGA | CD83 | TCGA |
| PATL2 | TCGA | PIP4K2A | TCGA | IL13 | TCGA |
| KMO | TCGA | HCLS1 | TCGA | TYROBP | TCGA |
| HIST2H3C | TCGA | SLFN12L | TCGA | LAT | TCGA |
| AC112229.3 | TCGA | REELD1 | TCGA | CHRNA5 | TCGA |
| TM4SF19 | TCGA | TRIM22 | TCGA | C2orf70 | TCGA |
| TNFRSF13B | TCGA | ZNF208 | TCGA | GMFG | TCGA |
| FGR | TCGA | CD27 | TCGA | FCGR2B | TCGA |
| BANK1 | TCGA | PLD4 | TCGA | PLEKHO1 | TCGA |
| BMF | TCGA | CD1D | TCGA | ICAM3 | TCGA |
| SCIMP | TCGA | PTCRA | TCGA | ZNF154 | TCGA |
| TDGF1 | TCGA | BIN2 | TCGA | SH3TC2 | TCGA |
| PRF1 | TCGA | CORO1A | TCGA | OXER1 | TCGA |
| TEX101 | TCGA | LRRC25 | TCGA | RASGRP4 | TCGA |
| TXK | TCGA | ITGB7 | TCGA | HIST1H2BD | TCGA |
| P2RX5-TAX1BP3 | TCGA | SCML4 | TCGA | KCNK1 | TCGA |
| NKG7 | TCGA | C12orf42 | TCGA | KCNIP2 | TCGA |
| PARVG | TCGA | KRT79 | TCGA | CXorf65 | TCGA |
| CD68 | TCGA | KLHL6 | TCGA | TNMD | TCGA |
| ADAD2 | TCGA | PSG5 | TCGA | HCST | TCGA |
| KRT72 | TCGA | GIMAP8 | TCGA | NIN | TCGA |
| LILRB1 | TCGA | P2RX7 | TCGA | AOAH | TCGA |
| PZP | TCGA | AJAP1 | TCGA | CKMT1A | TCGA |
| PTPN6 | TCGA | CLEC17A | TCGA | DTHD1 | TCGA |
| PAX5 | TCGA | ACSM3 | TCGA | SPDEF | TCGA |
| CNR2 | TCGA | OIT3 | TCGA | S1PR5 | TCGA |
| FCRL1 | TCGA | ADAMTS10 | TCGA | ZNF736 | TCGA |
| ZC3H12D | TCGA | CEP85L | TCGA | ITGA2B | TCGA |
| POU2F2 | TCGA | IKZF1 | TCGA | NLRP12 | TCGA |
| SOWAHD | TCGA | CD79B | TCGA | IL18BP | TCGA |
| ARHGAP25 | TCGA | CLNK | TCGA | LIPF | TCGA |
| CIITA | TCGA | FCER2 | TCGA | LYPD1 | TCGA |
| ADGRE1 | TCGA | CPN2 | TCGA | TACC2 | TCGA |
| HBA2 | TCGA | NPL | TCGA | CD300LG | TCGA |
| KLRK1 | TCGA | DNAJC5B | TCGA | P2RY10 | TCGA |
| WDFY4 | TCGA | MS4A7 | TCGA | AQP9 | TCGA |
| LILRB5 | TCGA | FNBP1 | TCGA | CD4 | TCGA |
| TSPAN32 | TCGA | SIGLEC14 | TCGA | ZNF486 | TCGA |
| IRF8 | TCGA | MPEG1 | TCGA | MUC13 | TCGA |
| CD300LB | TCGA | ROBO4 | TCGA | PLIN2 | TCGA |
| CD72 | TCGA | LAT2 | TCGA | KLRG1 | TCGA |
| CETP | TCGA | ARRB2 | TCGA | TMEM273 | TCGA |
| PDE2A | TCGA | SLAMF6 | TCGA | SLC25A45 | TCGA |
| TLX1 | TCGA | BMP2K | TCGA | RBP7 | TCGA |
| SLC5A10 | TCGA | ITGA4 | TCGA | TLCD1 | TCGA |
| TMEM217 | TCGA | NCF4 | TCGA | AGR2 | TCGA |
| TMC8 | TCGA | SCNN1G | TCGA | RUNX3 | TCGA |
| TMEM131L | TCGA | GGTLC1 | TCGA | SPN | TCGA |
| ITGAL | TCGA | NLRC4 | TCGA | KIF21B | TCGA |
| MATK | TCGA | MICAL1 | TCGA | DNAH1 | TCGA |
| MMP25 | TCGA | APBB1IP | TCGA | ABCB4 | TCGA |
| KCNJ10 | TCGA | CAMP | TCGA | HIST2H2BE | TCGA |
| CD244 | TCGA | ATP1A4 | TCGA | ARHGAP28 | TCGA |
| IL18RAP | TCGA | LAPTM5 | TCGA | TDRD10 | TCGA |
| CHI3L1 | TCGA | PTK2B | TCGA | COL19A1 | TCGA |
| KLRC4 | TCGA | GTSF1L | TCGA | STEAP2 | TCGA |
| FHOD1 | TCGA | RGS19 | TCGA | RFX4 | TCGA |
| KBTBD8 | TCGA | TNFRSF9 | TCGA | IGSF6 | TCGA |
| SSH2 | TCGA | DTX1 | TCGA | MAB21L4 | TCGA |
| EOMES | TCGA | CD48 | TCGA | IL16 | TCGA |
| LILRA4 | TCGA | TNFAIP8L2 | TCGA | STX16-NPEPL1 | TCGA |
| NPY5R | TCGA | GPR65 | TCGA | TOX3 | TCGA |
| HK3 | TCGA | ATM | TCGA | CD300E | TCGA |
| CD180 | TCGA | PDE1B | TCGA | CCDC200 | TCGA |
| PHOSPHO1 | TCGA | SLA | TCGA | C1QTNF9B | TCGA |
| KRT73 | TCGA | SEPT6 | TCGA | TMEM26 | TCGA |
| SASH3 | TCGA | AIF1 | TCGA | C1QA | TCGA |
| C2orf92 | TCGA | TTC16 | TCGA | LTA | TCGA |
| VAMP1 | TCGA | AIM2 | TCGA | IQANK1 | TCGA |
| PLEK | TCGA | ALAS2 | TCGA | ABHD17C | TCGA |
| ACAP1 | TCGA | LST1 | TCGA | IL12A | TCGA |
| CD52 | TCGA | LSP1 | TCGA | MYBPH | TCGA |
| PPP1R16B | TCGA | HLA-DMB | TCGA | VCAM1 | TCGA |
| CD300A | TCGA | YPEL1 | TCGA | MANSC1 | TCGA |
| PLCG2 | TCGA | NFAM1 | TCGA | NEURL3 | TCGA |
| BTK | TCGA | LRCOL1 | TCGA | EBI3 | TCGA |
| MPIG6B | TCGA | PLEKHO2 | TCGA | EPHB3 | TCGA |
| COL4A3 | TCGA | PCDH11X | TCGA | FASLG | TCGA |
| IKZF3 | TCGA | SIGLEC10 | TCGA | MLPH | TCGA |
| CYTH4 | TCGA | RETN | TCGA | FAM153A | TCGA |
| RASGRP3 | TCGA | RGL4 | TCGA | ETS1 | TCGA |
| ANKRD33B | TCGA | KCNAB2 | TCGA | SIRPD | TCGA |
| EXOC3L1 | TCGA | ARRDC5 | TCGA | PLEKHA6 | TCGA |
| KEL | TCGA | APOBEC3A | TCGA | DPPA4 | TCGA |
| CARD8 | TCGA | CXCL6 | TCGA | CARD9 | TCGA |
| MCOLN2 | TCGA | PIK3R5 | TCGA | OPRL1 | TCGA |
| HBB | TCGA | DOCK10 | TCGA | SLC15A3 | TCGA |
| ANGPTL6 | TCGA | BTN2A2 | TCGA | LYVE1 | TCGA |
| LY86 | TCGA | FAM53B | TCGA | FCER1G | TCGA |
| ANKRD44 | TCGA | CYFIP2 | TCGA | MEFV | TCGA |
| UNC45B | TCGA | DNHD1 | TCGA | AGER | TCGA |
| SPATA13 | TCGA | GPSM3 | TCGA | GNG7 | TCGA |
| BTLA | TCGA | MAP3K8 | TCGA | CAPN8 | TCGA |
| CDHR1 | TCGA | SIGLEC7 | TCGA | PXT1 | TCGA |
| DCSTAMP | TCGA | AC008878.3 | TCGA | CSF1R | TTD/TCGA |
| GDPD2 | TCGA | FCRL4 | TCGA | NQO1 | TTD/TCGA |
| MARCH1 | TCGA | PTPRC | TCGA | PSCA | TTD/TCGA |
| MAP4K1 | TCGA | ARHGAP4 | TCGA | CD19 | TTD/TCGA |
| FCAMR | TCGA | CREB5 | TCGA | MS4A1 | TTD/TCGA |
| PRKCB | TCGA | STAC3 | TCGA | CSF3R | TTD/TCGA |
| TNFRSF13C | TCGA | THEMIS2 | TCGA | TLR6 | TTD/TCGA |
