# Supplementary material for: A network pharmacology approach to reveal the pharmacological targets and biological mechanism of compound kushen injection for treating pancreatic cancer based on WGCNA and in vitro experiment validation
Source: Chin Med. 2021 Nov 22;16:121. doi: 10.1186/s13020-021-00534-y (PMC8607619; doi:10.1186/s13020-021-00534-y)
Supplement: Supplementary file 5 — Additional file 5: Table S3. Information of potential key targets in CKI-PC PPI network. [file 13020_2021_534_MOESM5_ESM.docx]

**Table S3.** Information of potential key targets in CKI-PC PPI network.

| **Target** | **Protein name** | **degree** | **betweenness** | **closeness** |
| --- | --- | --- | --- | --- |
| AKT1 | RAC-alpha serine/threonine-protein kinase | 31 | 0.17543392 | 0.64893617 |
| MAPK1 | mitogen-activated protein kinase 1 | 30 | 0.07964646 | 0.62244898 |
| CCNB1 | cyclin-B1 | 27 | 0.10730538 | 0.60396040 |
| MAPK3 | mitogen-activated protein kinase 3 | 26 | 0.04916655 | 0.59803922 |
| EGFR | epidermal growth factor receptor | 21 | 0.03328178 | 0.59223301 |
| STAT3 | signal transducer and activator of transcription 3 | 22 | 0.04828252 | 0.58653846 |
| PPP2CA | serine/threonine-protein phosphatase 2A catalytic subunit alpha isoform | 19 | 0.08076367 | 0.56481481 |
| CDC25C | M-phase inducer phosphatase 3 | 20 | 0.07305721 | 0.56481481 |
| EGF | pro-epidermal growth factor | 25 | 0.03307446 | 0.56481481 |
| PTPN1 | tyrosine-protein phosphatase non-receptor type 1 | 16 | 0.02585208 | 0.56481481 |
| CCNA2 | cyclin-A2 | 23 | 0.03073655 | 0.55454545 |
| AURKA | aurora kinase A | 23 | 0.03979766 | 0.54954955 |
| BIRC5 | baculoviral IAP repeat-containing protein 5 | 21 | 0.03263644 | 0.53508772 |
| CDK1 | cyclin-dependent kinase 1 | 24 | 0.03087434 | 0.52586207 |
| JAK1 | janus kinase 1 | 19 | 0.02126397 | 0.50000000 |
